# An arrhythmogenic metabolite in atrial fibrillation

**DOI:** 10.1186/s12967-023-04420-z

**Published:** 2023-08-24

**Authors:** Julia Krause, Alexander Nickel, Alexandra Madsen, Hamish M. Aitken-Buck, A. M. Stella Stoter, Jessica Schrapers, Francisco Ojeda, Kira Geiger, Melanie Kern, Michael Kohlhaas, Edoardo Bertero, Patrick Hofmockel, Florian Hübner, Ines Assum, Matthias Heinig, Christian Müller, Arne Hansen, Tobias Krause, Deung-Dae Park, Steffen Just, Dylan Aïssi, Daniela Börnigen, Diana Lindner, Nele Friedrich, Khaled Alhussini, Constanze Bening, Renate B. Schnabel, Mahir Karakas, Licia Iacoviello, Veikko Salomaa, Allan Linneberg, Hugh Tunstall-Pedoe, Kari Kuulasmaa, Paulus Kirchhof, Stefan Blankenberg, Torsten Christ, Thomas Eschenhagen, Regis R. Lamberts, Christoph Maack, Justus Stenzig, Tanja Zeller

**Affiliations:** 1 University Center of Cardiovascular Science, Department of Cardiology, University Heart and Vascular Center Hamburg, University Medical Center Hamburg-Eppendorf, Martinistrasse 52, 20246 Hamburg, Germany; 2 DZHK (German Centre for Cardiovascular Research), Partner Site Hamburg/Kiel/Lübeck, Hamburg, Germany; 3 Comprehensive Heart Failure Center, University Clinic Würzburg, Würzburg, Germany; 4 Institute of Experimental Pharmacology and Toxicology, University Medical Center Hamburg-Eppendorf, Hamburg, Germany; 5 Department of Physiology, HeartOtago, School of Biomedical Sciences, University of Otago, Dunedin, New Zealand; 6 Department of Cardiology, University Heart and Vascular Center Hamburg, Hamburg, Germany; 7 Institute of Food Chemistry, University of Münster, Münster, Germany; 8 Institute of Computational Biology, Helmholtz Zentrum München, Munich, Germany; 9 Department of Informatics, Technical University Munich, Munich, Germany; 10 Molecular Cardiology, Department of Internal Medicine II, University of Ulm, Ulm, Germany; 11 Institute of Clinical Chemistry and Laboratory Medicine, University Medicine Greifswald, Greifswald, Germany; 12 DZHK (German Centre for Cardiovascular Research), Partner Site Greifswald, Greifswald, Germany; 13 Department of Thoracic and Cardiovascular Surgery, University Clinic Würzburg, Würzburg, Germany; 14 Department of Intensive Care Medicine, University Medical Center Hamburg-Eppendorf, Hamburg, Germany; 15 Department of Epidemiology and Prevention, IRCCS Neuromed, Pozzilli, Italy; 16 Department of Medicine and Surgery, Research Center in Epidemiology and Preventive Medicine (EPIMED), University of Insubria, Varese, Italy; 17 Finnish Institute for Health and Welfare, Helsinki, Finland; 18 Center for Clinical Research and Prevention, Bispebjerg and Frederiksberg Hospital, Capital Region of Denmark, Copenhagen, Denmark; 19 Department of Clinical Medicine, Faculty of Health and Medical Sciences, University of Copenhagen, Copenhagen, Denmark; 20 Cardiovascular Epidemiology Unit, Institute of Cardiovascular Research, University of Dundee, Dundee, UK; 21 Institute of Cardiovascular Sciences, University of Birmingham, Birmingham, UK; 22 Present Address: Department of Cardiology and Angiology, Faculty of Medicine, University Heart Center Freiburg-Bad Krozingen, Medical Center – University of Freiburg, University of Freiburg, 79106 Freiburg, Germany

**Keywords:** Metabolites, Acyl-carnitine, Atrial fibrillation, Translational medicine, Engineered heart tissue

## Abstract

**Background:**

Long-chain acyl-carnitines (ACs) are potential arrhythmogenic metabolites. Their role in atrial fibrillation (AF) remains incompletely understood. Using a systems medicine approach, we assessed the contribution of C18:1AC to AF by analysing its in vitro effects on cardiac electrophysiology and metabolism, and translated our findings into the human setting.

**Methods and results:**

Human iPSC-derived engineered heart tissue was exposed to C18:1AC. A biphasic effect on contractile force was observed: short exposure enhanced contractile force, but elicited spontaneous contractions and impaired Ca^2+^ handling. Continuous exposure provoked an impairment of contractile force. In human atrial mitochondria from AF individuals, C18:1AC inhibited respiration. In a population-based cohort as well as a cohort of patients, high C18:1AC serum concentrations were associated with the incidence and prevalence of AF.

**Conclusion:**

Our data provide evidence for an arrhythmogenic potential of the metabolite C18:1AC. The metabolite interferes with mitochondrial metabolism, thereby contributing to contractile dysfunction and shows predictive potential as novel circulating biomarker for risk of AF.

**Supplementary Information:**

The online version contains supplementary material available at 10.1186/s12967-023-04420-z.

## Background

Atrial fibrillation (AF) affects up to 2% of the general population and remains a severe public health burden due to complications even on optimal therapy [1, 2]. The pathology of AF is complex and still incompletely understood. In addition to structural, contractile, and electrical features [3–5], metabolic alterations contribute to the pathogenesis of AF [6, 7], and vice versa AF can induce cardio-metabolic changes [8].

The metabolism of long-chain fatty acids in the mitochondria represents the main energy source for cardiac work [9]. The uptake of long-chain fatty acids from the cytoplasm into mitochondria is achieved by forming long-chain acyl-carnitines (ACs) [10]. These metabolites are readily accessible for quantification in the circulation and are routinely analysed in new-born screenings to detect inherited metabolic disorders [11]. The origin of circulating ACs can be both cellular release and dietary uptake. Recently, studies showed an association between AC metabolism and cardiovascular diseases, such as heart failure [12–14], suggesting a potential role of long-chain ACs as circulating biomarkers. The role of ACs in AF has not been extensively researched. With regard to the underlying mechanism, data suggest that long-chain ACs interfere with energy metabolism, Ca^2+^ handling, and membrane surfaces, suggesting a role for arrhythmia development [15, 16]. Yet, the exact mechanisms are unknown and the majority of these experiments was restricted to ventricular cardiomyocytes.

Here, we used a systems medicine approach and assessed the contribution of the metabolite C18:1AC to AF pathology by evaluating its i) metabolic and contractile properties using state-of-the-art in vitro models and ii) by translation of key findings into human cohorts. We revealed that C18:1AC compromises mitochondrial respiration and has profound effects on the contractile function of cardiac tissues leading to arrhythmic contractions. Translation in the human setting confirmed AF-dependent differences in C18:1AC serum concentration and metabolism.

## Methods

A detailed description of the methods is provided in the supplement.

### Stem cell culture and cardiac differentiation

Stem cell culture and atrial differentiation were performed as described previously [17].

### Engineered heart tissue (EHT) generation and cultivation

Generation and analysis by video-optical recording of EHT generated from human induced pluripotent stem cell-derived cardiomyocytes were performed as previously described [17, 18].

### EHT exposure to metabolites

EHT experiments with the metabolites C18:1AC (1 µM, 5 µM, 10 µM, 25 µM) and C3AC (as one control group, 25 µM) were performed in serum-free medium. EHTs were exposed to different concentrations of C18:1AC up to 25 µM as described previously [10, 13, 19]. Additional controls were exposed to solvent only. The compounds were dissolved in H_2_O according to manufacturer’s instructions. Experiments with electrical field stimulation were carried out with an electrical stimulator in Tyrode’s solution containing different Ca^2+^ concentrations [20].

For human ventricular EHT (vEHT), previous studies had revealed a positive force frequency relationship between beating rates of 0.75 to 2.5 Hz [21]. Thus, to avoid potential masking of positive inotropic effects at higher frequencies and consequently maximal force, frequency of vEHT was decreased to ~ 0.5 Hz (Additional file 1: Fig. S2a) by adding ivabradine (300 nM, selective I_f_ current inhibitor). Ivabradine could not be used for atrial EHT (aEHT, 4 Hz spontaneous frequency), as previous experiments had revealed a strong negative inotropic response.

### C18:1AC detection by mass spectrometry

For high pressure liquid chromatography electrospray ionization tandem mass spectrometry (HPLC–ESI–MS/MS), human EHTs were dissociated by enzymatic digestion with papain, and fractionated according to the fractionation kit’s manufacturer’s instructions (Cell Signaling #9038). All material (EHTs, EHT fractions, sub-cellular fractions) was subjected to acetonitrile-based lipid extraction. The supernatant was collected and frozen at − 80 °C before HPLC–ESI–MS/MS.

### Inotropy measurements

Experiments with EHTs were conducted in Tyrode’s solution with a Ca^2+^ concentration (0.6–1.5 mM) which was individually titrated to evoke submaximal force defined as 30–70% of maximal force in both human vEHT and aEHT, respectively. The effect of the metabolite and other substances on contractile force was measured under electrical stimulation as previously described [20]. Isoprenaline, ryanodine, cyclopiazonic acid (CPA), and isobutylmethylxanthine (IBMX) were prepared according to the manufacturer’s instructions.

### Action potential measurements in human EHT models

Action potentials (APs) were measured in EHTs by sharp microelectrode as previously described [22].

### Experiments in 2D culture with murine ventricular and hiPSC-derived cardiomyocytes

Changes in intracellular Ca^2+^ and mitochondrial membrane potential in C18:1AC exposed murine ventricular cardiomyocytes or hiPSC-derived cardiomyocytes were assessed with fluorescent dyes in 2D culture [23, 24].

### Human atrial trabeculae dissection and metabolite exposure

After human atrial trabeculae preparation, the tissues were superfused with 37 °C 1.5 mM Ca^2+^ Krebs–Henseleit Buffer (KHB) and field stimulated at 1 Hz (suprathreshold voltage, 5 ms duration). Spontaneous contraction propensity was assessed [25]. Trabeculae force was normalised to muscle cross sectional area as determined by width × thickness × π.

### Isolation of human and murine mitochondria

Mitochondria were isolated from adult mouse ventricles (12–15 weeks old) as well as left atrial appendages from patients undergoing surgery (ethics approval no. 143/17-sc, University Clinic Würzburg) using an established method optimised for small tissue samples [26, 27]. All animal procedures were conforming to the guidelines of Directive 2010/63/EU of the European Parliament on the protection of animals used for scientific purposes and were approved by the local animal ethics committee. Animals were euthanized by cervical dislocation.

### Measurements of mitochondrial respiration and determination of ΔΨ_m_ in isolated mitochondria and atrial EHT

Mitochondrial oxygen consumption was measured polarographically at 37 °C using a Clark oxygen electrode (Oxygraph, Hansatech) [23, 27], and an Oroboros Oxygraph-2 k device, using 400 µg mitochondria or 2 EHTs for each respirometry chamber [27, 28]. Mitochondrial membrane potential (ΔΨ_m_) was simultaneously probed using 1 µM of the potentiometric fluorescent dye tetramethylrhodamine methyl ester (TMRM) and Smart Fluo-Sensor Green (Thermo Fisher Scientific). DatLab V7.4 software (Oroboros Instruments) was used for data acquisition and analysis.

### Measurements of nicotinamide adenine dinucleotide phosphate

NAD(P)H was quantified using a fluorescence plate reader (Infinite 200 Pro, Tecan). All experiments were conducted in respiration buffer as described previously, with 320 µg mitochondria per well in duplicates (excitation at 340 nm, emission at 450 nm) in 200 µL reaction volume [27]. Pyruvate/malate (5 mM each), ADP (1 mM), cyanide (2.5 mM), oligomycin/DNP (1.2 µM/ 250 µM) were added as indicated. Data were calculated as fluorescence intensity normalised to basal fluorescence (F/F_0_).

### Human cohort studies

Serum and tissue samples of 3 different studies were used:

(i) Tissue samples obtained from a collective of patients called Würzburg cohort including patients in sinus rhythm (n = 29), with paroxysmal AF (PAF, n = 10), and with sustained AF (SAF, n = 6, Additional file 1: Table S1),
(ii) a patient cohort undergoing cardiac surgery, here called the New Zealand cohort, again consisted of patients in sinus rhythm (n = 28), with PAF (n = 13), and with SAF (n = 11, Additional file 1: Table S2).
(iii) The population-based case-cohort set of the BiomarCaRE (Biomarker for Cardiovascular Risk Assessment in Europe) consortium [29, 30] comprised 1,437 incident AF cases and 7,591 non-cases, followed up for a period of 20 years (Additional file 1: Tables S3 and S4).

All studies were approved by the respective local ethics boards (FINRISK97: 82/2001, Moli-sani: Prot.pdc.P.99/A.931/03-128-04/C:E./2004, DanMONICA: 1980-272-2 1051NE/bt 2-16-2/43(85) KA 90238, New Zealand: #LRS12-01-001AM1-AM17, Würzburg: 143/17-sc).

### Metabolome data

By mass spectrometry, serum concentrations of 186 metabolites from 6 different compound classes were analysed, using the AbsoluteIDQ p180 kit (Biocrates). After quality control, 141 and 163 metabolites were used for analysis in BiomarCaRE and the New Zealand cohort, respectively.

### Statistical analysis

Experimental data were analysed and visualised with GraphPad Prism 6 and 8 software. Data in the text are expressed as mean ± SEM. In BiomarCaRE, associations with time-to-AF were assessed for log-transformed metabolite concentrations using Cox proportional hazard models [31]. The analyses were adjusted for body mass index (BMI), systolic blood pressure (SBP), antihypertensive treatment, diabetes, total cholesterol, sex, daily smoking, study centre, and age at baseline examination. For the screening in the New Zealand diseased cohort, differential concentrations of long-chain ACs were analysed using a linear regression model adjusting for age, sex, BMI, SBP, diastolic blood pressure (DBP), diabetes, smoking, and ejection fraction.

## Results

### C18:1AC induces a positive inotropic effect in human EHT and atrial trabeculae

To characterise the effect of long-chain C18:1AC on AF pathology we assessed the short-term effect of increasing C18:1AC concentrations on contractility in human ventricular engineered heart tissue (vEHT), atrial EHT (aEHT), and human atrial trabeculae.

**Fig. 1:**
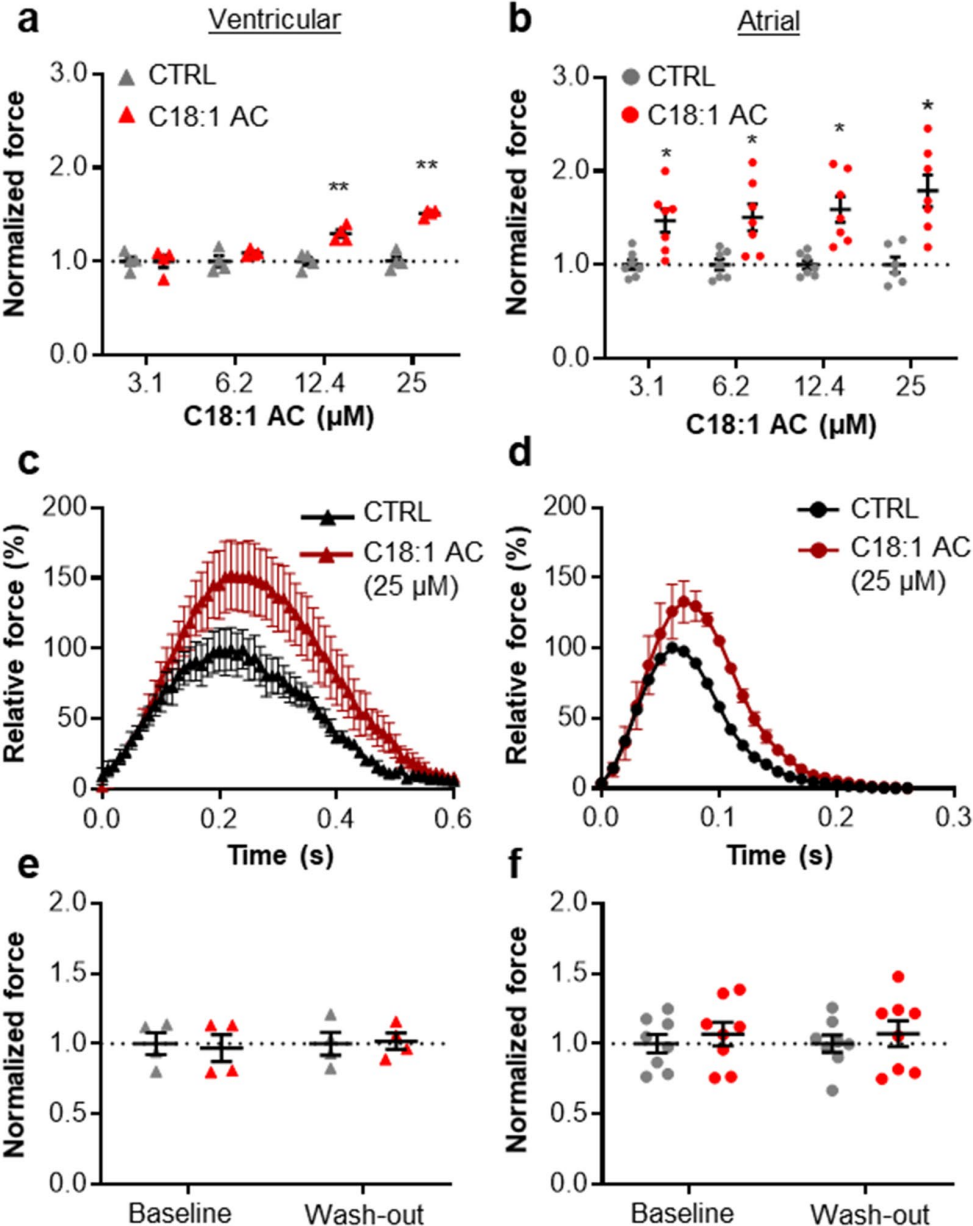
Positive inotropic effect of C18:1AC on contractility of human vEHT (left panel) and aEHT (right panel).** a** Human vEHT (0.5 Hz; 0.8 mM calcium, n = 4/1 per group) and **b** aEHT (3.6 Hz, 1.4–1.5 mM calcium; n = 8/1 per group, 2 independent experiments) exposed to increasing concentrations of C18:1AC for 20 min each under electrical stimulation (mean ± SEM, two-way ANOVA plus Bonferroni’s post-test for multiple comparisons, *p < 0.05, **p < 0.01 vs. vehicle control). Force normalised to baseline and corresponding vehicle controls (dotted line). Average contraction peaks of **c** vEHT (paced at 0.5 Hz) and **d** aEHT (3.6 Hz) after incubation with 25 µM C18:1AC for 20 min (mean ± SEM). Force normalised to corresponding vehicle control for **e** vEHT and **f** aEHT before and 24 h after washing out C18:1AC. Numbers for EHT experiments refer to total number of EHTs/number of batches

At extracellular Ca^2+^ concentrations evoking submaximal force, both vEHT and aEHT reacted to C18:1AC exposure with a concentration-dependent increase in force (Fig. 1a, b, Additional file 1: Fig. S2). C18:1AC at a concentration of 25 µM for 20 min induced an increase in force by 51.3 ± 1.9% at 0.5 Hz in vEHT and 79.0 ± 17.0% at 3.6 Hz in aEHT (Fig. 1a–d, Additional file 1: Fig. S3). After 24 h of wash-out, no differences in force remained between C18:1AC exposure and control groups in both vEHT and aEHT (Fig. 1e, f, Additional file 1: Fig. S2). Furthermore, 20 min of C18:1AC incubation (25 µM) led to a modest but significant prolongation of the action potential duration at 90% repolarization (APD_90_; from 123.2 ± 11.2 ms to 133.0 ± 11.2 ms) in aEHT, whereas in vEHT no pronounced alterations in electrophysiology were observed after C18:1AC exposure (Additional file 1: Fig. S4).

**Fig. 2:**
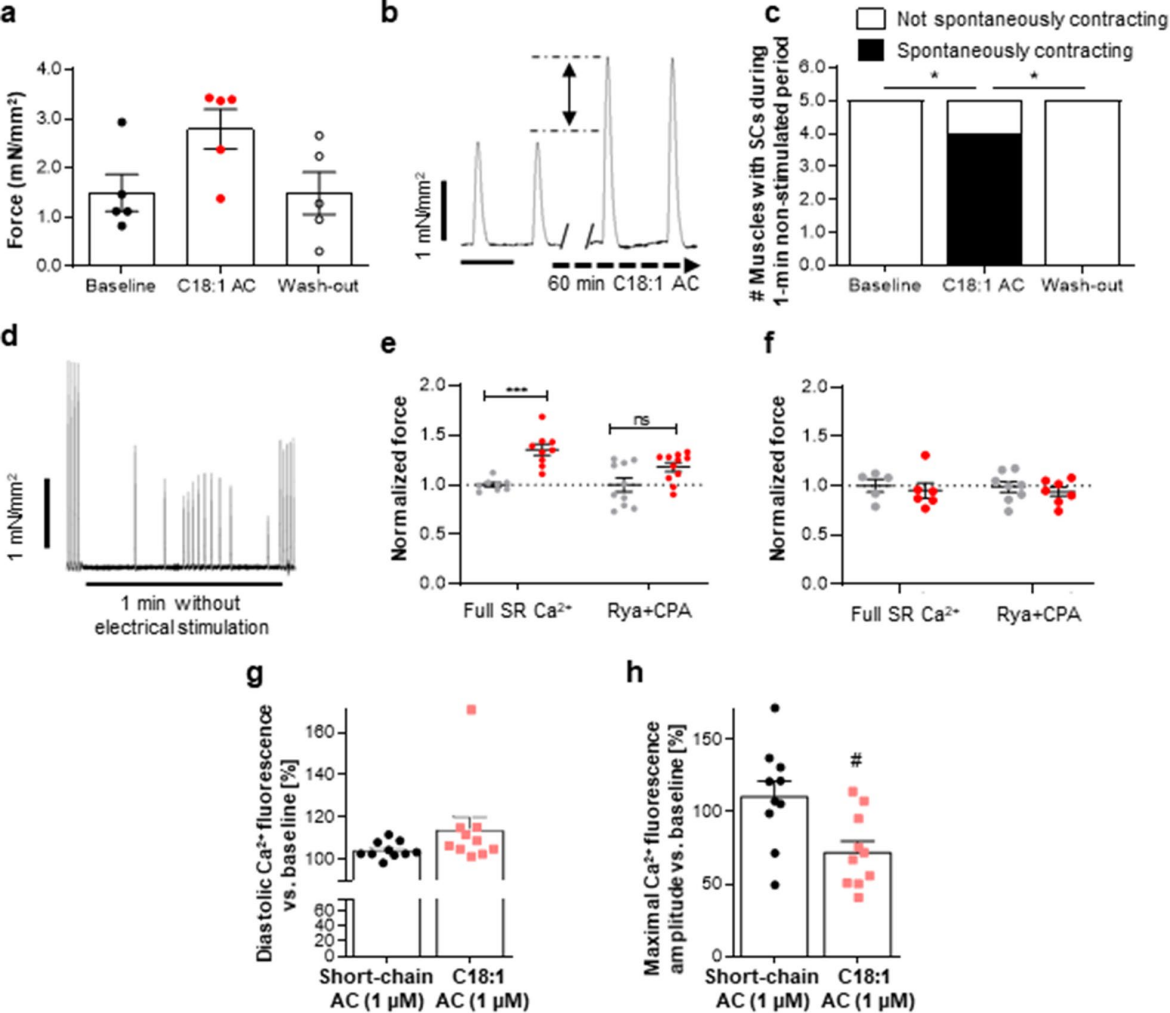
Arrhythmogenic effect of C18:1AC on contractility of human atrial trabeculae and on single murine ventricular cardiomyocytes.** a** Force of human right atrial trabeculae after 1 h exposure to 25 µM C18:1AC and 30 min wash-out (mean ± SEM, n = 5/5, number of trabeculae/number of patients). **b** Representative contraction peaks before and after 1 h of superfusion with C18:1AC at 25 µM. **c** Number of trabeculae with spontaneous contractions (SC) during 1 min interval without electrical stimulation after C18:1AC exposure and **d** representative traces with each peak representing one contraction (n = 5, Χ^2^ tests, *p < 0.05). **e** Inotropic effect of C18:1AC on human atrial EHT (3.6 Hz, 1.2–1.5 mM Ca^2+^, n = 8–12/2 per group (number of EHTs/batches), three technically independent experiments) under electrical stimulation in the presence of full SR Ca^2+^ and after treatment with ryanodine and cyclopiazonic acid to deprive the SR of Ca^2+^ (Rya + CPA, mean ± SEM, two-way ANOVA plus Bonferroni’s post-test for multiple comparisons, ***p < 0.001 vs. vehicle control). Force normalised to baseline and corresponding vehicle controls (dotted line). **f** Force normalised to corresponding vehicle control 24 h after washing out C18:1AC. SR = sarcoplasmic reticulum. **g** Murine ventricular cardiomyocytes were loaded with the calcium-dye Indo-AM to determine cytosolic Ca^2+^ and were stimulated at 1 Hz. After 120 s, C18:1 or short-chain AC were washed in for 420 s. The increase in diastolic Ca^2+^ concentration was more pronounced in C18:1AC group compared to the short-chain AC group. **h** Systolic Ca^2+^ transients were decreased in the C18:1AC group, compared to the point of origin

To compare these results to inotropy in primary human tissue, atrial trabeculae were electrically stimulated at 1 Hz and exposed to 25 µM C18:1AC. Similar to our observations in human EHT, C18:1AC induced an increase in force by 86.3% ± 26.9% after 1 h, which was reversed after wash-out (Fig. 2a, b). In addition, C18:1AC (25 µM) evoked spontaneous contractions in human atrial trabeculae, indicating an arrhythmic potential of C18:1AC (Fig. 2c, d). This effect was again reversed after wash-out of C18:1AC (Fig. 2c).

### C18:1AC induces arrhythmia and interferes with intracellular Ca^2+^ handling

Data from our in vitro models provided evidence for a positive inotropic effect of C18:1AC. A pivotal mechanism of positive inotropic effects includes changes in Ca^2+^ homeostasis [21]. Therefore, we explored an influence of long-chain ACs on intracellular Ca^2+^ in different tissues and cell culture models.

As a measure for sarcoplasmic reticulum (SR) Ca^2+^ leak and Ca^2+^ load, we evaluated the post-pause force in human atrial trabeculae exposed to C18:1AC and in control tissues after a 1-min break of electrical stimulation. Atrial trabeculae exposed to C18:1AC displayed a greater reduction in post-pause force compared to control conditions (vehicle only, − 34.8 ± 12.8%, vs. − 16.4 ± 25.0%, Additional file 1: Fig. S5), which was reversed after wash-out. The reduction in force argues for an SR Ca^2+^ leak rather than SR Ca^2+^ accumulation, which was more pronounced under C18:1AC exposure. To further explore the influence of SR Ca^2+^ in atrial cardiomyocytes, aEHTs were additionally treated with ryanodine (1 µM) and cyclopiazonic acid (10 µM) to deprive the SR from Ca^2+^ prior to C18:1AC exposure [32]. Under these conditions, the positive inotropic effect of C18:1AC was significantly reduced but not completely abolished (+ 35.3 ± 5.6% with SR Ca^2+^, + 17.8 ± 4.3% with reduced SR Ca^2+^, Fig. 2e, Additional file 1: Fig. S6). This effect was again reversible after 24 h of wash-out (Fig. 2f). The combination of the β-adrenergic agonist isoprenaline (300 nM) and the phosphodiesterase inhibitor IBMX (10 µM) without C18:1AC was used as positive control to increase force independently of SR Ca^2+^. A strong positive inotropic effect was indeed observed, irrespective of whether the SR was Ca^2+^ deprived or not (Additional file 1: Fig. S6). As a next step, this effect of C18:1AC on intracellular Ca^2+^ handling was explored in single murine cardiomyocytes and in atrial hiPSC-derived cardiomyocytes in 2D culture. Exposure of murine ventricular cardiomyocytes in 2D culture to ≥ 1 µM C18:1AC induced arrhythmia as defined by extra beats during electrical stimulation, which was not observed in cells exposed to the control short-chain C3AC (Additional file 1: Fig. S7). Between 5 µM and 25 µM C18:1AC induced Ca^2+^ overload immediately followed by mitochondrial membrane collapse resulting in cell death (Additional file 1: Fig. S8). In hiPSC-derived cardiomyocytes, only one concentration of C18:1AC, 1 µM, was applied. These cells were left to beat spontaneously and reacted to C18:1AC treatment with a higher arrhythmic index compared to hiPSC-derived cardiomyocytes treated with the same concentration of the control short-chain C3AC (Additional file 1: Fig. S7).

To further assess an influence of C18:1AC on systolic and diastolic Ca^2+^, Ca^2+^ concentrations were measured in adult murine cardiac myocytes, which were electrically stimulated at 1 Hz for 420 s and incubated with either 1 µM of C18:1AC or short-chain C3AC. Cells incubated with C18:1AC exhibited a more pronounced increase in diastolic Ca^2+^ concentration (Fig. 2g and Additional file 1: Fig. S9) and a reduction in systolic Ca^2+^-transient amplitude (Fig. 2h and Additional file 1: Fig. S9). Conversely, no changes were found in Ca^2+^ decay kinetics, the SR Ca^2+^ load measured after addition of 10 mM caffeine, and the fractional Ca^2+^ release (Additional file 1: Fig. S9).

Lastly, we investigated if suspected changes in Ca^2+^ handling could be attributed to an influence of C18:1AC treatment on expression, degradation or post-translational modifications of important players in Ca^2+^ cycling. To this end, we treated atrial hiPSC-derived cardiomyocytes in 2D-culture with 25 µM of C18:1AC for 20 min. Western blot analysis of important Ca^2+^ handling related proteins revealed no differences between treated and non-treated conditions (Additional file 1: Fig. S10).

### Interference of C18:1AC with cardiac contractility

**Fig. 3:**
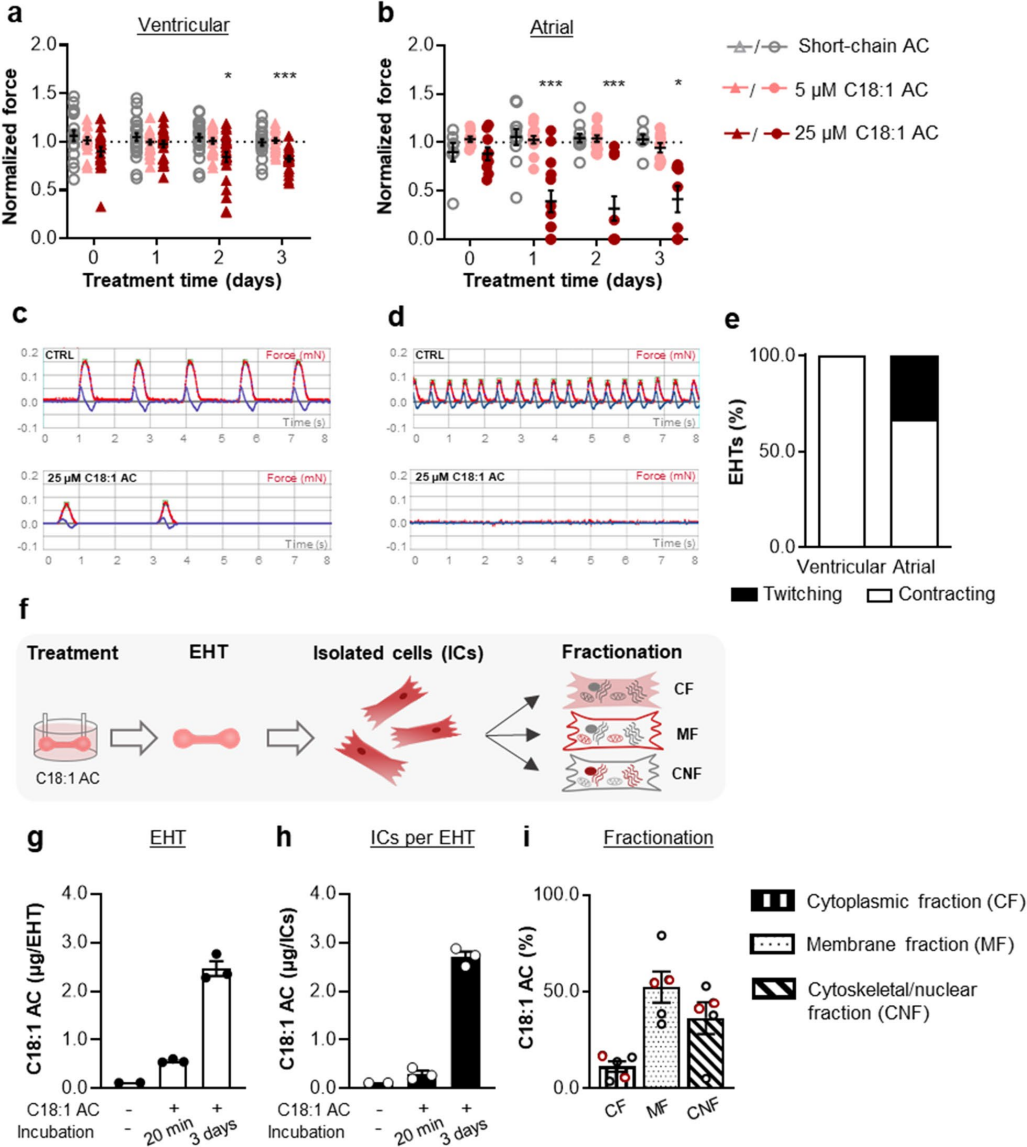
Time-dependent effect of C18:1AC on human vEHT and aEHT. Force of human **a** vEHT and **b** aEHT in response to exposure to 25 µM short-chain C3AC (vEHT n = 20–24/5, aEHT n = 7–11/3), 5 µM C18:1AC (vEHT n = 18–23/5, aEHT n = 9–14/3) and 25 µM C18:1AC (vEHT n = 18–24/5, aEHT n = 8–13/3) over 3 days. Exposure to 25 µM C18:1AC induced a decrease in contractile force in vEHT and aEHT in a distinct manner. Parameters normalised to corresponding vehicle control (dotted line). Two-way ANOVA plus Dunnett’s post-test for multiple comparisons vs. control, *p < 0.05, ***p < 0.001. Mean ± SEM. Segments of representative original contractility recording traces of **c** vEHTs and **d** aEHTs exposed to solvent only or 25 µM C18:1AC for 3 days (red line = force in mN, blue line = velocity in mN/s). The recording trace of the 25 µM C18:1AC exposed aEHT represents a “twitching” contractile phenotype. **e** Human EHTs were grouped according to contractile phenotype after two weeks of exposure to 25 µM of C18:1AC. Grouped as “contraction” (deflection of posts) and “twitching/wobbling” (convulsions with no deflection of posts). Only aEHTs, but not vEHTs displayed twitching phenotypes (vEHT n = 24/5, aEHT n = 13/3). Numbers in brackets show total number of EHTs/number of batches. **f** Graphical illustration of the protocol to trace C18:1AC in EHT. C18:1AC in **g** whole aEHTs (per whole EHT: 1 million cells and fibrin matrix, n = 3 EHTs) and **h** hiPSC-derived cardiomyocytes from dissociated aEHTs (per dissociated EHT: 1 million cells, n = 3 EHTs) after exposure to 25 µM of C18:1AC for 20 min or 3 days. Untreated aEHTs served as controls (n = 2 EHTs per group). **i** Relative C18:1AC amount from different sub-cellular fractions after isolation of cardiomyocytes from aEHT and vEHT followed by sub-cellular fractionation after 3 days of exposure to 10 µM of C18:1AC (aEHT n = 2 indicated by red circles, vEHT n = 3)

To address possible contractile consequences of longer-term C18:1AC exposure, rather than minutes to a few hours, as described above, we extended the incubation time of vEHT and aEHT with C18:1AC to 3 days and regularly assessed contractility. Controls were exposed to i) solvent only or ii) to 25 µM C3AC. Exposure to 5 µM of C18:1AC did not significantly compromise the contractility of vEHT and aEHT (Fig. 3a, b, Additional file 1: Fig. S11). However, in vEHT and aEHT exposed to 25 µM C18:1AC, a progressive decline of beating rate and contractile force was observed over time. Human vEHT showed a gradual decline in both parameters, with a drop in frequency (− 24.7 ± 3.5%, Additional file 1: Fig. S11) and normalised force (− 15.8 ± 5.2%, Fig. 3a) already after 2 days of exposure. In contrast, aEHT exhibited an even more rapid decline of both parameters. This decline in frequency (− 32.6 ± 11%, Additional file 1: Fig. S11) and normalised force (− 61.0 ± 11.2%, Fig. 3b) was detectable already after 1 day. A 3-day pre-treatment with 1 µM C18:1AC did not protect aEHT treated with 25 µM C18:1AC from the decline in normalised force (Additional file 1: Fig. S11c). In addition, the contraction behaviour in a proportion of treated aEHTs was characterised by a twitching phenotype resulting in non-detectable force (Fig. 3c–e).

### C18:1AC accumulates within cardiomyocytes and progressively interferes with contractility

To confirm the presence of C18:1AC within EHTs after exposure, the concentration of C18:1AC was measured by HPLC–ESI–MS/MS at different time points (Fig. 3 f, Additional file 1: Fig. S12). First, we assessed the cumulative amount of C18:1AC in intact EHTs and in hiPSC-derived cardiomyocytes isolated from EHTs after exposure to 25 µM of C18:1AC for either 20 min or 3 days. Compared to untreated control EHTs, we observed an accumulation of C18:1AC in both intact EHT and in isolated cells over time (Fig. 3g, h, Additional file 1: Fig. S12). To test whether C18:1AC integrates into cellular membrane systems, we fractionated hiPSC-derived cardiomyocytes from EHT into different compartments including membrane, cytoplasm and cytoskeleton/nucleus. Indeed, qualitative results showed that C18:1AC was mainly detectable in the cytoskeletal and membrane fraction (Fig. 3i).

### Concentration-dependent effect of C18:1AC on mitochondrial respiration

**Fig. 4:**
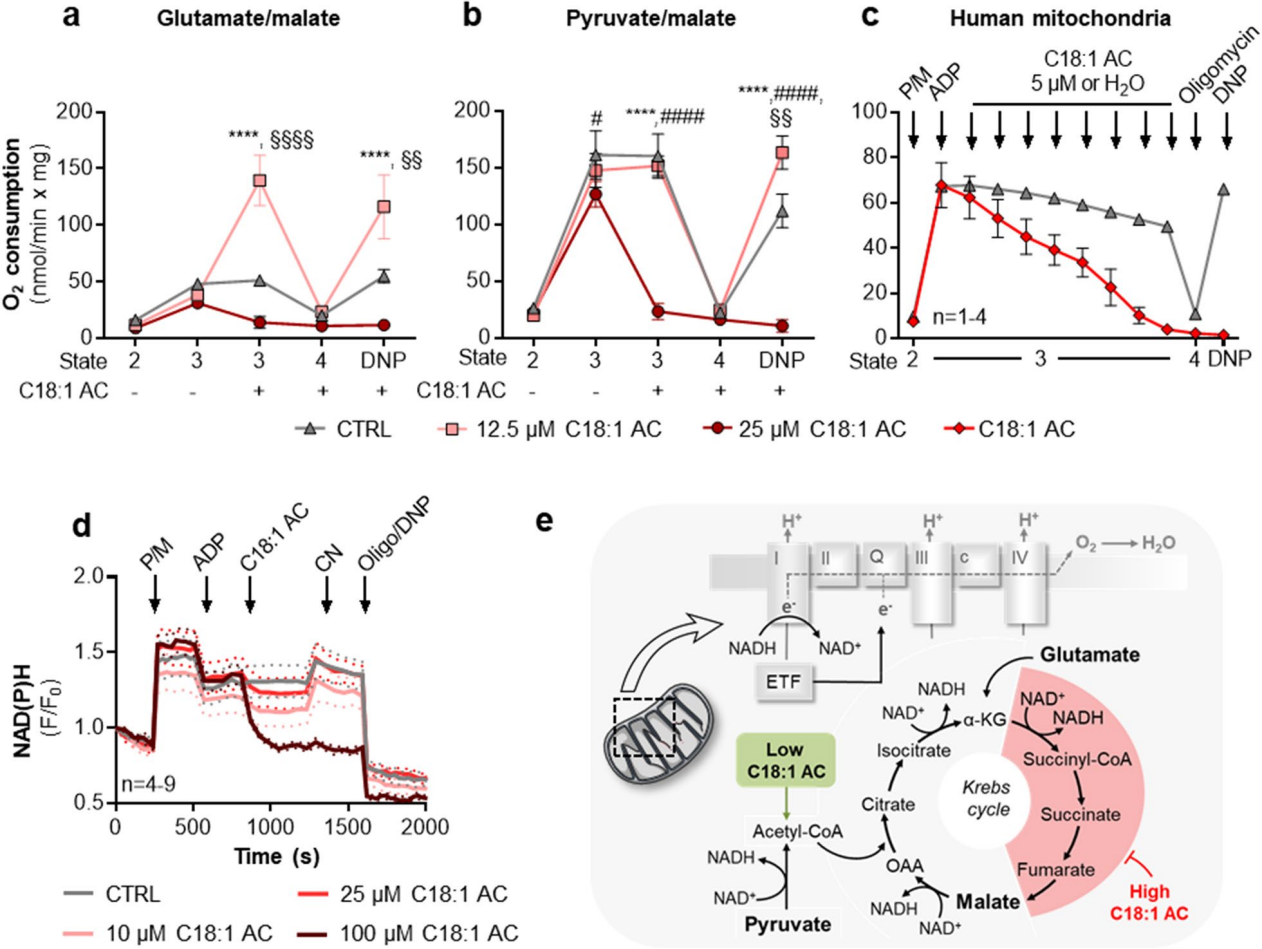
Biphasic effect of C18:1AC on mitochondrial respiration. Respiration of murine cardiac mitochondria supplied with **a** glutamate/malate (G/M) or **b** pyruvate/malate (P/M) as substrates, exposed to 12.5 or 25 µM C18:1AC (n = 3–7 mouse hearts). Mitochondrial respiration states: State 2 = basal state without compounds; State 3 = activated state with ATP production; State 4 = no ATP production. Respiratory state 3 obtained by stimulating ATP synthase with saturating (1 mM) ADP. State 4 induced by oligomycin (1.2 µM) to block ATP synthase and 2,4-dinitrophenol (DNP) to uncouple respiration. **c** Sequential addition of C18:1AC (5 µM each) during P/M respiration of human atrial mitochondria. **d** NAD(P)H autofluorescence of murine cardiac mitochondria supplied with P/M and ADP (1 mM), addition of 10, 25, or 100 µM C18:1AC. Cyanide (CN) added to inhibit complex IV, reducing NAD(P)H, preventing oxidation by the electron transport chain. Oligomycin and DNP added to induce complete oxidation of NAD(P)H. **e** Scheme of Krebs cycle and electron transport chain and potential mechanism of inhibition by C18:1AC. Two-way ANOVA plus Bonferroni´s post-test for multiple comparisons: ****p < 0.0001 for C18:1AC 12.5 µM vs. 25 µM; ^§§^p < 0.01, ^§§§§^p < 0.0001 for C18:1AC 12.5 µM vs. control; ^#^p < 0.05, ^####^p < 0.0001 for C18:1AC 25 µM vs. control; *n* number of hearts. *α-KG* α-ketoglutarate, *AU* arbitrary units, *ADP* adenosine diphosphate, *DNP* dinitrophenol, *ETF* electron transfer flavoprotein, *OAA* oxaloacetate, *TMRM* tetramethylrhodamine methyl ester

The healthy heart is highly dependent on sufficient energy supply with ATP via mitochondrial respiration. Therefore, we investigated whether and how C18:1AC affects mitochondrial respiration, both at a moderate (10–12.5 µM) and very high concentration (25 µM). Mitochondria isolated from mouse ventricular cardiomyocytes and human atrial tissue in sinus rhythm and AF were used, and the effects of C18:1AC on ADP-stimulated respiration and mitochondrial membrane potential (ΔΨ_m_) were assessed. To additionally investigate substrate-dependent C18:1AC effects, different energy substrates (glutamate/malate (G/M) or pyruvate/malate (P/M)) were used (Fig. 4 e).

In isolated murine mitochondria, at a moderate C18:1AC concentration (12.5 µM) respiration was already maximally activated when P/M was used as substrate. Upon respiration with G/M as substrate, addition of C18:1AC acutely stimulated respiration, showing that C18:1AC can be used as additional substrate. In contrast, at high concentrations (25 µM) C18:1AC almost completely abolished respiration and dissipated ΔΨ_m_ regardless of the substrate (Fig. 4a, b, Additional file 1: Fig. S13). To exclude irreversible mitochondrial membrane damage by high C18:1AC, murine mitochondria were pre-incubated with 25 µM C18:1AC followed by a resuspension in AC-free respiration buffer. After resuspension, mitochondrial respiration recovered, indicating that C18:1AC did not irreversibly damage mitochondrial membranes (Additional file 1: Fig. S14a). In an assessment of mitochondrial respiration directly from EHT, tissues which had been exposed to 25 µM of C18:1AC for 4 days displayed markedly lower respiration with P/M/G as substrate compared to controls. Additional pre-treatment with 1 µM of C18:1AC for 3 days had no further impact on mitochondrial respiration (Additional file 1: Fig. S14b).

To discriminate between possible effects of C18:1AC on either the Krebs cycle or the electron transport chain, the redox state of NAD(P)H was assessed. For this, we took into account that NAD(P)H is first reduced by the Krebs cycle and is subsequently oxidised by the electron transport chain. Addition of 25 µM C18:1AC to murine mitochondria led to a decrease in NAD(P)H autofluorescence (Fig. 4d), reflecting oxidation of the NAD(P)H pool (Fig. 4e). Treatment with an inhibitor of pyruvate dehydrogenase (CPI-613) led to a similar response (Additional file 1: Fig. S14c, d). Taken together, these results indicate an inhibition of NAD(P)H provision to the respiratory chain (i.e. Krebs Cycle) by C18:1AC rather than blockade of the electron transport chain per se*.* Similar to the observations in murine mitochondria we also observed a C18:1AC concentration-dependent inhibition of respiration when using human mitochondria isolated from the left atrial myocardium (Fig. 4c, Additional file 1: Fig. S13).

**Fig. 5:**
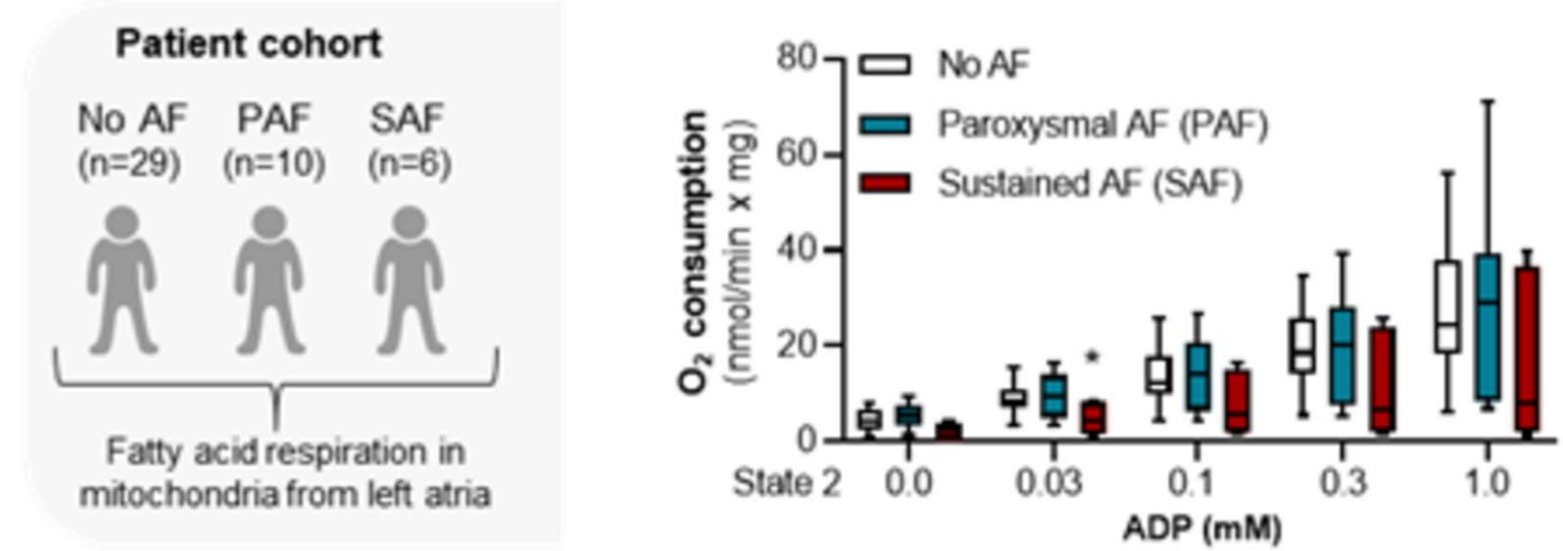
Altered mitochondrial respiration in patients with sustained AF. Left: Schematic illustration of the patient cohort (Würzburg cohort) used to investigate AF-dependent changes in mitochondrial metabolism. Right: Comparison between oxygen consumption rate of isolated human atrial mitochondria from patients in sinus rhythm, with PAF or SAF using C18:1AC (10 µM), palmitoyl-CoA and malate as substrates. Mitochondrial respiration was reduced in samples from patients with SAF (median, whiskers: min to max, two-way ANOVA plus Dunnett’s post-test for multiple comparisons, *p < 0.05 for SAF vs. no AF)

We further analysed AF-dependent changes in mitochondrial respiration and assessed whether isolated mitochondria from AF patients react differently to low C18:1AC as substrate (10 µM). Mitochondria were isolated from patients of the Würzburg cohort in sinus rhythm (n = 29), paroxysmal AF (PAF, n = 10) or sustained AF (SAF, n = 6, Additional file 1: Table S1), without overt differences in serum C18:1 AC concentration (Additional file 1: Fig. S14e). In mitochondria from patients in sinus rhythm and PAF, we observed an ADP-dependent increase in respiration. In contrast, respiration rates were lower in mitochondria from patients with SAF, suggesting an AF-associated deficit in C18:1AC metabolism (Fig. 5).

### Association between C18:1AC serum concentrations and incidence of atrial fibrillation

Our in vitro data suggested arrhythmogenic properties of C18:1AC and an impairment of Ca^2+^ handling and metabolism by C18:1AC. Additionally, in samples of patients with AF, our results showed an AF-associated deficit in C18:1AC metabolism. These observations suggested that C18:1AC serum concentrations might be elevated in AF-patients, either contributing to the pathogenesis of AF, as a consequence of AF or both, creating a vicious cycle.

Therefore, we further explored whether concentrations of circulating C18:1AC were changed in AF patients and quantified C18:1AC serum concentrations in patients from the New Zealand cohort comprising 13 PAF and 11 SAF cases as well as 28 control subjects in sinus rhythm (Additional file 1: Table S2). An association between SAF and elevated serum concentration of C18:1AC was observed (ß: 0.3029, FDR < 0.05) whereas no significant association was found for PAF.

**Fig. 6:**
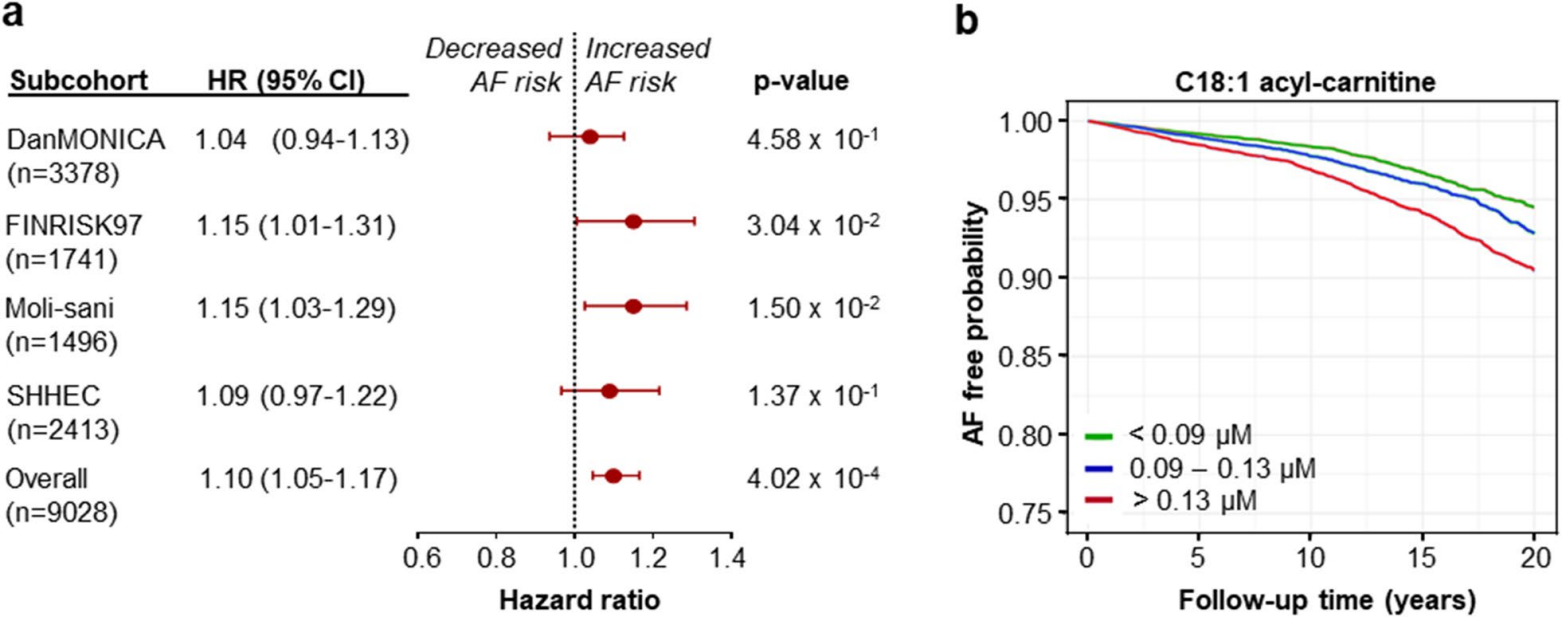
Association between circulating C18:1AC concentration and AF risk.** a** Association between acyl-carnitine (AC) serum concentration and atrial fibrillation (AF) risk over time analysed in the overall BiomarCaRE case-cohort set consisting of four different community-based cohorts (DanMONICA, FINRISK97, Moli-sani, SHHEC). Cox regression analyses were performed for each study centre, adjusted for multiple cardiovascular risk factors. Hazard ratios (HRs) and 95-percent confidence interval (95% CI) of the association between C18:1AC serum concentration and AF development shown for each cohort study. HRs are reported for 1 standard deviation (SD) increase. **b** Kaplan–Meier curve of AF free probability over time depending on C18:1AC serum concentration assessed in the BiomarCaRE case-cohort set

As these data indicated an association of circulating C18:1AC with AF, we further investigated the predictive potential of its serum concentrations for the development of future AF. For this purpose, metabolite data of the BiomarCaRE AF case-cohort set [30], comprising 1,437 incident AF cases and 7,591 non-cases were analysed (Additional file 1: Tables S3A, B, S4). Figure 6 a shows a significant association between serum C18:1AC concentrations and incidence of AF with an HR of 1.10 (CI 95% 1.05–1.17, p < 0.0004). Higher serum concentrations of C18:1AC were associated with lower AF free probability over a follow-up period of up to 20 years as illustrated by Kaplan–Meier curves (Fig. 6b), indicating the potential of C18:1AC as novel circulating biomarker for the prediction of AF.

## Discussion

**Fig. 7:**
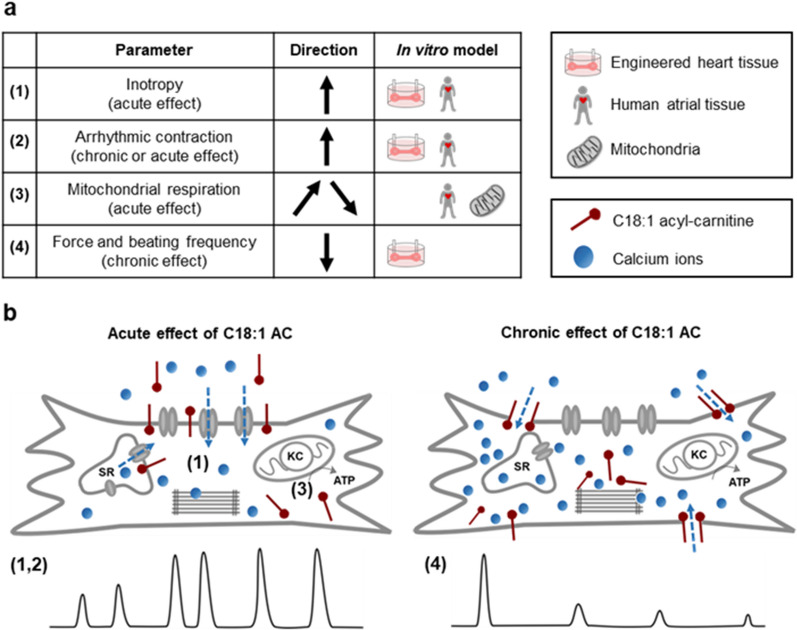
Summary and putative mechanism of action of C18:1AC. Elevated serum concentrations of the long-chain acyl-carnitine C18:1AC were identified to be associated with incident and prevalent atrial fibrillation (AF), and **a** the arrhythmogenic potential of C18:1AC was experimentally assessed using different in vitro models. **b** Our experiments support the idea that the bipolar metabolite C18:1AC can interact with cellular membranes and thereby influences associated receptors and ion channels. We propose that C18:1AC concentrations can increase intracellular Ca^2+^ concentration which induces a positive inotropic effect (1) but also arrhythmic events (2, illustrated as irregular contraction peaks). C18:1AC evoked a biphasic effect on mitochondrial respiration (3) characterised by stimulation at lower and inhibition at higher concentrations. Based on our identification of an impaired C18:1AC metabolism in patients with sustained AF, we hypothesise that long-chain ACs contribute to the maintenance of AF as follows: Reduced oxygen supply during AF results in impaired mitochondrial β-oxidation leading to accumulation of long-chain ACs, which in turn affects mitochondrial metabolism and contractility further. We suggest that long-time exposure to high C18:1AC concentrations, for instance during ischemic conditions, results in progressive cellular accumulation of the metabolite and consequently rather non-specific effects like impaired cardiac contractility (4). *ATP* adenosine triphosphate, *KC* Krebs cycle, *SR* sarcoplasmic reticulum

Cardio-metabolic alterations are contributors to the pathogenesis of AF. This study aimed to assess the contribution of the long-chain C18:1AC to AF in depth. We unraveled (i) an arrhythmogenic potential of C18:1AC through interference with cardiac Ca^2+^ handling, (ii) C18:1AC-dependent changes in mitochondrial substrate utilization. In a translational approach, (iii) we confirmed our findings in human mitochondria of AF-patients and showed the potential of circulating C18:1AC as novel biomarker for AF risk prediction (Fig. 7).

### Long-chain C18:1 acyl-carnitine induces a positive inotropic effect

Abnormalities in intracellular Ca^2+^ handling are crucial contributors to ventricular and atrial arrhythmia [33]. An acute exposure to C18:1AC induced a concentration-dependent positive inotropic effect in vEHT and aEHT. When increasing the time of exposure, a decrease in contractile force was observed, thus adding an important temporal aspect of the effect to previous observations [16, 26]. Notably, in contrast to vEHT, the majority of aEHTs additionally presented some signs of more irregular beating behaviour, further suggesting an arrhythmogenic effect of the C18:1AC and thus a special role for the pathogenesis of AF. Moreover, the short-chain (control-) AC did not compromise contractility, supporting the argument that the activity of C18:1AC depends on its chain length and possibly saturation status [10]. By definition, long-chain ACs have a carbon chain length ≥ 14 and an amphiphilic character, suggesting that the mechanism of action involves the integration of C18:1AC into cellular membrane systems [34]. Indeed, the membrane and cytoskeletal compartments of cardiomyocytes in EHT accumulated the highest proportion of C18:1AC compared to the cytoplasmic fractions in our experiments. Moreover, with continuous exposure we quantified increasing concentrations of C18:1AC in hiPSC-derived cardiomyocytes over time, arguing for progressive accumulation of the metabolite. This might also explain the time-dependent impairment of contractility in the EHT model, suggesting a rather non-specific negative inotropic effect of the metabolite at accumulation, in contrast to the defined positive inotropic mechanism with short-term exposure. This reversible inotropic effect of C18:1AC seems to be based on specific changes in Ca^2+^ sensitivity, intracellular Ca^2+^ homeostasis and further inotropy modulating mechanisms.

### Long-chain C18:1 acyl-carnitine triggers spontaneous contractions

One fundamental mechanism of arrhythmia induction is aggravated electrical activity caused e.g. by delayed afterdepolarization due to high intracellular Ca^2+^ concentrations [35]. Previously, long-chain ACs (C16 AC) were shown to elicit Ca^2+^ overload in cardiomyocytes via controversial and probably multifactorial mechanisms. Proposed actions of the long-chain C16 AC comprise impairment of Ca^2+^, Na^+^, and K^+^ ion transport across both the plasma membrane and sarcoplasmic reticulum (SR) membrane [10, 34]. In our experiments, C18:1AC triggered spontaneous contractions in human atrial trabeculae. This could possibly be explained by an SR Ca^2+^ leak, which may have contributed to the spontaneous contractions e.g. via an increased Na^+^/Ca^2+^ exchanger current resulting in the initiation of an AP [36]. The observed decrease in post-pause force of atrial trabeculae exposed to C18:1AC again argues in this direction, as the decline could indicate Ca^2+^ release from SR storage, which would be less filled at the time of the first beat post-pause, due to AC-induced increased leakage. Arguing further in this direction, the positive inotropic effect of the metabolite was markedly reduced after eliminating SR Ca^2+^ in human aEHT. Consistently, other studies using ventricular cardiomyocytes as well as HEK293 cells exposed to long-chain ACs had revealed a ryanodine receptor-mediated Ca^2+^ leak [13, 36, 37]. Yet, the positive inotropic effect of C18:1AC we observed was not completely abolished after depriving the SR of Ca^2+^, which indicates the involvement of additional mechanisms [10, 13, 38]. Our novel findings from the fluorescence-based Ca^2+^ quantification in 2D-cultured cardiomyocytes argue in the same direction. In mouse ventricular cardiomyocytes (paced at 1 Hz), treatment with C18:1AC led to an increase in diastolic Ca^2+^ concentration and a decrease in Ca^2+^-transient amplitude, but with no effect on Ca^2+^ decay kinetics or SR Ca^2+^ load. Increased diastolic Ca^2+^-concentration can lead to an activation of Ca^2+^-dependent enzymes like the CaMKII delta C, which is known to provoke arrhythmia [39]. We repeated these experiments also in hiPSC-derived cardiomyocytes, but only observed an increased variability in beating frequency in the C18:1AC treated cells. To account for their high spontaneous beating frequency, the cells were left to beat spontaneously. We observed a progressive decline in frequency during the experiment (more pronounced in the cells exposed to C18:1AC), which might have potentially masked effects on Ca^2+^ handling together with a retained high SERCA activity. The greater potency of C18:1AC in single cells might be attributable to a high relation of the amount of freely available C18:1AC compared to a small overall volume of lipophilic cellular membrane, resulting in a faster accumulation of the compound within the single cardiomyocytes compared to multicellular in vitro models. Our experimental data support the hypothesis that C18:1AC interferes with automaticity of atrial cardiomyocytes, a pivotal mechanism of cardiac arrhythmia, partially by causing cytoplasmic Ca^2+^ overload.

### A link between AF, mitochondrial respiration and long-chain C18:1 acyl-carnitine

In the healthy heart, the high energy turnover required to maintain cellular homeostasis during contraction and for Ca^2+^ handling is primarily met by fatty acid β-oxidation. Compared to glucose metabolism this yields more ATP but also requires more oxygen per ATP [40]. Thus, potential changes of cardiac energy metabolism in favour of β-oxidation might become detrimental to cardiac efficiency and oxygen consumption under conditions of high energy demand [41]. Our observations in both intact aEHT and in isolated mitochondria, the generators of long-chain ACs, are in line with findings of previous studies documenting interference with mitochondrial respiration by other long-chain ACs [42–44]. Exceeding previous experiments, our data indicate that, at low concentrations, C18:1AC can be oxidised by cardiac mitochondria, while higher concentrations inhibit respiration by hindering the Krebs-cycle-dependent supply of electrons to the transport chain, both in intact aEHT and in isolated mitochondria. As low concentrations of C18:1AC could be metabolized and as additional evidence suggested a possible metabolic adaptation to ACs, we evaluated the effect of pre-treatment of aEHT with a low concentration (1 µM) of C18:1AC before exposure to a high concentration (25 µM) on mitochondrial respiration. However, our data rather argue for an additional incorporation or at least no sufficient protection from the impact of the high C18:1AC treatment. In light of these considerations, we aimed to assess the clinical relevance by investigating a possible link between cardio-metabolic alterations, AC concentrations and actual AF. In a situation of impaired oxidative metabolism, e.g. during myocardial ischemia, the resulting block of fatty acid-based respiration induces mitochondrial accumulation of long-chain AC [45]. Thus, we first compared mitochondrial fatty acid respiration between tissue samples from patients in sinus rhythm or with AF. Notably, respiration of mitochondria supplied with C18:1AC was reduced in patients with sustained AF (SAF) but not paroxysmal AF (PAF). Compared to PAF, the irregular contractions during SAF result in progressive pathophysiological changes which can manifest e.g. in an altered cardiac metabolism [46]. These results, together with the small sample size, may explain our observation that mitochondrial respiration was dependent on AF state, but not directly on prior C18:1AC serum concentration.

In accordance with the hypothesis that disturbed mitochondrial respiration during AF results in AC accumulation, we detected an association of higher serum concentrations of long-chain ACs in SAF but not PAF patients in an independent human cohort. This possible link between long-chain ACs and AF was further supported as higher C18:1AC serum concentrations were associated with the development of AF in our large population-based cohort study. This suggested the potential use of acyl-carnitine C18:1AC as circulating biomarker for AF risk. Although at serum concentrations of up to 0.2 µM, C18:1AC might probably not directly interfere with cardiac metabolism, the lipophilic nature of ACs suggests high tissue binding. Indeed, tissue concentrations reported in animal models were higher [47] and our own preliminary investigations to quantify C18:1AC in cardiac tissue samples collected during autopsies indicate C18:1AC concentrations of up to 30 µM (data not shown). Furthermore, ACs might accumulate in mitochondria and membranes due to e.g. impaired fatty acid oxidation, as observed in our patients with sustained AF and in patients developing post-operative AF as well as in in vitro models [48, 49]. Our current metabolic in vitro and in vivo results suggest that high concentrations of circulating C18:1AC in AF can alter substrate preference and mitochondrial oxidative metabolism. Inhibition of mitochondrial respiration and subsequent decrease in the ATP/ADP ratio can affect diverse energy-dependent processes, such as Ca^2+^ handling, potentially culminating in impaired contractility or arrhythmia [50]. In line with this, it was recently described that C18:1AC interferes with the heart function of zebrafish by disturbing mitochondrial function [16].

### Limitations

We acknowledge some limitations. First, further study is warranted to unravel the precise mechanisms underlying the reduced mitochondrial respiration. In this regard, a direct correlation between an altered cardiac metabolism and the circulating AC pool needs to be validated, ideally e.g. by quantifying arterial-to-venous differences in C18:1AC serum concentrations across the heart in patients with and without AF or other heart disease. Additionally, as our findings and findings from the literature suggest a class effect of long-chain ACs, studies evaluating further long-chain ACs are needed to directly confirm such a class effect. Second, it remains to be elucidated whether there is a direct link between the C18:1AC-dependent changes in mitochondrial respiration and Ca^2+^ handling. Third, in new-born infants suffering from fatty acid metabolism disorders concentrations of circulating C18:1AC up to 21 µM are measured [51]. Though this is in the range of the concentration we employed in vitro, the commonly observed serum concentrations in AF patients are considerably lower. Nevertheless, we believe our experiments are mechanistically representative, as our data argue for membrane disturbances after progressive accumulation as a main mechanism of action. To achieve sufficient incorporation, higher concentrations are required in our short-term in vitro models. However, this discrepancy remains to be considered when interpreting our results. Lastly, as the serum concentrations of C18:1AC are only moderately elevated in subjects with increased AF risk, quantifying co-regulated compounds in parallel to C18:1AC might increase its value as a biomarker when combined into a biomarker panel.

In summary, our findings suggest that C18:1AC interferes with intracellular Ca^2+^ and mitochondrial metabolism, supporting its general arrhythmogenic potential. Translation into the human setting confirmed AF-dependent changes in C18:1AC metabolism and suggests C18:1AC as a novel circulating biomarker for AF risk prediction. Circulating C18:1AC and potentially other long-chain ACs may provide access to previously hidden cardio-metabolic information and be part of a newly discovered vicious circle in AF [37, 42]. In this circle, reduced oxygen supply results in impaired mitochondrial β-oxidation leading to accumulation of long-chain ACs. These, in turn, inhibit mitochondrial metabolism further, aggravating contractility further. Future research should be directed towards elucidating the metabolic mechanisms underlying cardiac arrhythmias in more detail, and whether metabolite panels may guide targeted AF therapies.

### Supplementary Information

**Additional file 1. Table S1.** Baseline characteristics of the Würzburg patient cohort. **Table S2.** Baseline characteristics of the New Zealand patient cohort. **Table S3.** **A** Baseline characteristics of the overall BiomarCaRE case-cohort study population. **B** Baseline characteristics of the subcohorts comprising the BiomarCaRE case-cohort study population. **Table S4**. Number of individuals included in the BiomarCaRE case-cohort set. **Fig. S1.** Differentiation of human induced pluripotent stem cells into ventricular and atrial cardiomyocytes. **Fig. S2.** Force-frequency relationship. **Fig. S3.** Short-term effect of C18:1AC on contractility of human EHT. **Fig. S4. **Action potentials of C18:1AC exposed human vEHT and aEHT. **Fig. S5.** Post-pause force of atrial trabeculae after C18:1AC exposure. **Fig. S6. **Positive inotropic effect of C18:1AC. **Fig. S7.** Arrhythmogenic potential of C18:1AC. **Fig. S8. **Influence of C18:1AC on calcium load and mitochondrial membrane potential in murine ventricular cardiomyocytes. **Fig. S9.** Influence of C18:1AC on systolic and diastolic calcium load in murine ventricular cardiomyocytes. **Fig. S10.** Western blots of calcium handling related proteins. **Fig. S11.** Time and concentration-dependent effect of C18:1AC on human vEHT and aEHT contractility (time course raw data). **Fig. S12.** Detection of C18:1AC in human EHT. **Fig. S13.** Biphasic effect of C18:1AC on mitochondrial respiration. **Fig. S14.** Influence of C18:1AC on mitochondrial respiration.

## Data Availability

The metabolomics data are available under restricted access, as they contain identifying participant information. Deposition in online repositories or controlled access repositories is not authorized by the patients’ consent. Access to the complete data including phenotypes can be obtained by any qualified researcher as part of an academic or industry collaboration. Co-authorship on resulting publications is required only if authorship criteria are fulfilled according to the guidelines of good scientific practice of the Deutsche Forschungsgemeinschaft (10.5281/zenodo.3923602). Requests including a formal research proposal indicating the use of data and planned analyses should be addressed to Tanja Zeller (t.zeller@uke.de) and will be processed within two weeks. Successful applications enable the unrestricted analysis of the data in the context of cardiovascular disease. All experimental data are available in the main text or the supplementary materials.
