# Supplementary material for: An arrhythmogenic metabolite in atrial fibrillation
Source: J Transl Med. 2023 Aug 24;21:566. doi: 10.1186/s12967-023-04420-z (PMC10464005; doi:10.1186/s12967-023-04420-z)
Supplement: Supplementary file 1 — Additional file 1. Table S1. Baseline characteristics of the Würzburg patient cohort. Table S2. Baseline characteristics of the New Zealand patient cohort. Table S3. A Baseline characteristics of the overall BiomarCaRE case-cohort study population. B Baseline characteristics of the subcohorts comprising the BiomarCaRE case-cohort study population. Table S4. Number of individuals included in the BiomarCaRE case-cohort set. Fig. S1. Differentiation of human induced pluripotent stem cells into ventricular and atrial cardiomyocytes. Fig. S2. Force-frequency relationship. Fig. S3. Short-term effect of C18:1AC on contractility of human EHT. Fig. S4. Action potentials of C18:1AC exposed human vEHT and aEHT. Fig. S5. Post-pause force of atrial trabeculae after C18:1AC exposure. Fig. S6. Positive inotropic effect of C18:1AC. Fig. S7. Arrhythmogenic potential of C18:1AC. Fig. S8. Influence of C18:1AC on calcium load and mitochondrial membrane potential in murine ventricular cardiomyocytes. Fig. S9. Influence of C18:1AC on systolic and diastolic calcium load in murine ventricular cardiomyocytes. Fig. S10. Western blots of calcium handling related proteins. Fig. S11. Time and concentration-dependent effect of C18:1AC on human vEHT and aEHT contractility (time course raw data). Fig. S12. Detection of C18:1AC in human EHT. Fig. S13. Biphasic effect of C18:1AC on mitochondrial respiration. Fig. S14. Influence of C18:1AC on mitochondrial respiration. [file 12967_2023_4420_MOESM1_ESM.docx]

Additional file 1: Material and figures

An arrhythmogenic metabolite in atrial fibrillation

Julia Krause^1,2^, Alexander Nickel^3^, Alexandra Madsen^2,4^, Hamish M. Aitken-Buck^5^, A. M. Stella Stoter^2,4^, Jessica Schrapers^2,4^, Francisco Ojeda^6^, Kira Geiger^3^, Melanie Kern^3^, Michael Kohlhaas^3^, Edoardo Bertero^3^, Patrick Hofmockel^3^, Florian Hübner^7^, Ines Assum^8,9^, Matthias Heinig^8,9^, Christian Müller^2,6^, Arne Hansen^2,4^, Tobias Krause^2,4^, Deung-Dae Park^10^, Steffen Just^10^, Dylan Aïssi^6^, Daniela Börnigen^6^, Diana Lindner^2,6$^, Nele Friedrich^11,12^, Khaled Alhussini^13^, Constanze Bening^13^, Renate B. Schnabel^2,6^, Mahir Karakas^2,14^, Licia Iacoviello^15,16^, Veikko Salomaa^17^, Allan Linneberg^18,19^, Hugh Tunstall-Pedoe^20^, Kari Kuulasmaa^17^, Paulus Kirchhof^2,6,21^, Stefan Blankenberg^2,6^, Torsten Christ^2,4^, Thomas Eschenhagen^2,4^, Regis R. Lamberts^5^, Christoph Maack^3^, Justus Stenzig^2,4✝^, Tanja Zeller^1,2✝*^

^✝^these authors contributed equally to this work

^1^University Center of Cardiovascular Science, University Heart and Vascular Center Hamburg, Hamburg, Germany; ^2^DZHK (German Centre for Cardiovascular Research), partner site Hamburg/Kiel/Lübeck, Hamburg; ^3^Comprehensive Heart Failure Center, University Clinic Würzburg, Würzburg, Germany; ^4^Institute of Experimental Pharmacology and Toxicology, University Medical Center Hamburg-Eppendorf, Hamburg, Germany; ^5^Department of Physiology, HeartOtago, School of Biomedical Sciences, University of Otago, Dunedin, New Zealand; ^6^Clinic of Cardiology, University Heart and Vascular Center Hamburg, Hamburg, Germany; ^7^Institute of Food Chemistry, University of Münster, Münster, Germany; ^8^Institute of Computational Biology, Helmholtz Zentrum München, München, Germany; ^9^Department of Informatics, Technical University Munich, München, Germany; ^10^Molecular Cardiology, Department of Internal Medicine II, University of Ulm, Ulm, Germany; ^11^Institute of Clinical Chemistry and Laboratory Medicine, University Medicine Greifswald, Greifswald, Germany; ^12^DZHK (German Centre for Cardiovascular Research), partner site Greifswald, Greifswald, Germany; ^13^Department of Thoracic and Cardiovascular Surgery, University Clinic Würzburg, Würzburg, Germany; ^14^Department of Intensive Care Medicine, University Medical Center Hamburg-Eppendorf, Hamburg, Germany; ^15^Department of Epidemiology and Prevention, IRCCS Neuromed, Pozzilli, Italy; ^16^Research Center in Epidemiology and Preventive Medicine (EPIMED), Department of Medicine and Surgery, University of Insubria, Varese, Italy; ^17^Finnish Institute for Health and Welfare, Helsinki, Finland; ^18^Center for Clinical Research and Prevention, Bispebjerg and Frederiksberg Hospital, Capital Region of Denmark, Denmark, Copenhagen; ^19^Department of Clinical Medicine, Faculty of Health and Medical Sciences, University of Copenhagen, Copenhagen, Denmark; ^20^Cardiovascular Epidemiology Unit, Institute of Cardiovascular Research, University of Dundee, Dundee, United Kingdom; ^21^Institute of Cardiovascular Sciences, University of Birmingham, Birmingham, United Kingdom, ^$^present address: Department of Cardiology and Angiology, University Heart Center Freiburg-Bad Krozingen, Medical Center – University of Freiburg, Faculty of Medicine, University of Freiburg, 79106 Freiburg, Germany

**Expanded materials and methods**

1. Stem cell culture and cardiac differentiation

Stem cell culture and differentiation were performed as described by Breckwoldt et al. (1). The human induced pluripotent stem cell (hiPSC) line UKEi003-C was established by the UKE Stem Cell Core Facility by reprogramming fibroblasts from the skin biopsy of a healthy individual with the Sendai virus-based CytoTune kit (Life Technologies). Cultivation and expansion of the hiPSC line was carried out in the presence of bFGF, TGFβ, dorsomorphin, and activin A. The differentiation of hiPSCs into ventricular and atrial cardiomyocytes was based on a three-step protocol starting with the induction of embryoid body (EB) formation as previously described (Figure S1 a and b) (1) (2). EB formation was initiated by constant stirring in spinner flasks for 24 h followed by mesoderm induction by treatment with BMP-4 (10 ng/mL, R&D Systems), activin A (3 ng/mL, R&D Systems), and bFGF (5 ng/mL, R&D Systems) for ventricular differentiation. For atrial differentiation, lower concentrations of BMP-4 (3 ng/mL) and activin A (2 ng/mL) were used. After three days, cardiac differentiation was initiated by inhibiting the WNT signalling pathway with XAV-939 (1 µM, Tocris). For atrial differentiation, the cardiac specification medium was additionally supplemented with 1 µM retinoic acid. Retinoic acid (Sigma) was dissolved in DMSO (50 mM, Sigma), further diluted in sterile water (100 µM), and stored at -20 °C. To obtain single hiPSC-derived cardiomyocytes, EBs were enzymatically dissociated with collagenase II (200 U/mL, Worthington) in Hank’s balanced salt solution without Ca^2+^/Mg^2+^ (Gibco) at the end of cardiac differentiation (day 17). The differentiation efficiency was determined by flow cytometry after staining for the cardiomyocyte marker cardiac troponin T (Miltenyi, #130-119-575; Figure S1 c). Only differentiation runs with >60% of clearly troponin T-positive cells were used.

1. Engineered heart tissue (EHT) generation and cultivation

Human ventricular EHT (vEHT) and atrial EHT (aEHT) was prepared as previously described (3). In brief, cardiomyocytes were suspended in DMEM medium (10% heat-inactivated foetal calf serum, 1% L-glutamine (Gibco), 1% penicillin/streptomycin (Gibco)) and mixed with fibrinogen (Sigma) and thrombin (Sigma). For one EHT 1 Mio cells were used. The EHT master mix was transferred into agarose casting moulds with two silicone mounting posts extending into each mould. After polymerization, EHTs adhered to the mounting posts and were transferred to and cultivated in standard EHT medium (DMEM with 10% heat-inactivated horse serum (Thermo Fisher Scientific), 1% penicillin/streptomycin, 10 µg/mL insulin (Sigma), and 33 µg/mL aprotinin (Sigma)) at 37 °C, 7% CO_2_, 40% O_2_ for up to 55 days. Contractility of EHTs was analysed by video-optical recording using custom-designed software (Consulting Team Machine Vision; CTMV) as described before (4). To account for different metabolite-induced phenotypes (contraction, inactivity, twitching), we defined the following criteria for analysis of the video-optical recording data: (i) In general, contractile parameters were only evaluated if the force reached a threshold of 0.01 mN. (ii) For inactive EHTs, which displayed no contraction during measurement, frequency was set to zero and the force was excluded from analysis. (iii) Twitching EHTs were characterised by visible movement without deflection of the posts, resulting in a contractile force below the threshold of 0.01 mN. For these EHTs, force was set to zero and the activity was excluded from analysis due to the visible but not quantifiable incoherent beating activity. Electrical stimulation was carried out with an electrical stimulator (Grass S88X, Natus Neurology Incorporated) at 2.5 V with biphasic pulses of 4 ms in Tyrode’s solution containing different Ca^2+^ concentrations.

1. Gene expression and histological analysis

Gene expression analysis was performed in atrial and ventricular hiPSC-derived cardiomyocytes after dissociation at day 17, as described by Lemme et al. (2). In brief, qPCR was carried out using HOT FIREPol EvaGreen qPCR mix (Solis BioDyne) with published primer sequences. For histological analysis, the EHTs were fixed in 4% formaldehyde overnight. Subsequent processing steps including embedding in paraffin, sectioning, and antigen retrieval (Tris-EDTA buffer). All processing steps and staining were performed at the Mouse Pathology Core Facility of the UKE. The paraffin sections were automatically stained using the Ventana BenchMark XT system (Roche). The following antibodies were used: MLC2A (Synaptic Systems 311 011) 1:75 and MLC2V (Synaptic Systems 310 111) 1:3000. The UltraView Universal DAB Detection Kit was used for staining with the secondary antibody and a BZ-X710 microscope (Keyence) for analysis.

1. EHT exposure to metabolites

Acyl-carnitines (ACs, propionyl-L-carnitine, C3AC, Sigma, #42602; oleoyl-L-carnitine, C18:1AC, Sigma, #597562) were dissolved in water according to the manufacturer’s instructions, aliquoted, and stored at -20 °C. Prior to the experiments, the medium was changed from standard EHT medium to serum-free medium (1% penicillin/streptomycin, 10 µg/mL insulin, 33 µg/mL aprotinin, 50 ng/mL hydrocortisone (Sigma), 0.5 ng/mL T3 (European Commission)) at an age of 18-22 days for EHTs, and baseline contractility measurement was performed. EHTs were subsequently assigned to different groups with similar average contractile force. AC supplemented medium was exchanged every day and video-optical recordings were performed after 1 h of incubation after each media change.

1. Inotropy measurements

The influence of ACs on inotropy was analysed in EHTs between day 41 and 47 of culture. Prior to experiments with the metabolite, extracellular Ca^2+^ was reduced by replacing the media with Tyrode’s solution containing 0.6 mM Ca^2+^ for vEHT and 1 mM Ca^2+^ for aEHT. When the EHTs reached a contractile force between 30-60% of their respective baseline force measured in 1.8 mM Ca^2+^, the wash-out procedure was stopped. Next, the EHTs were transferred into Tyrode’s solution with distinct submaximal Ca^2+^ concentrations for both models (vEHT: 0.6 mM Ca^2+^; aEHT: 1.4-1.5 mM Ca^2+^). The EHTs were allowed to equilibrate for 20 min before concentration-response experiments were performed with increasing concentrations of the long-chain AC or solvent only. Contractility was measured after 20 min of incubation with the AC in each respective concentration or other pharmacological reagents, under electrical stimulation as previously described. Average contraction peaks were generated from 15-20 individual contraction peaks in an automatic manner. Before these experiments, human vEHT was exposed overnight to 300 nM ivabradine (Sigma) to decrease the spontaneous beating frequency, enabling analysis of contractility at 0.5 Hz. Ivabradine was not used for aEHT since preliminary experiments had revealed a strong negative inotropic effect. As a result, experiments could only be performed at a lower frequency threshold of 3.6 Hz. EHTs not following pacing impulses were excluded from the analysis.

1. Human atrial trabeculae collection

The use of human myocardial tissue for this study was approved by the local ethics board (ethics #LRS12-01-001AM1-AM17) and in line with the Declaration of Helsinki. Samples from the right atrial appendage (RAA) were collected from consenting patients undergoing coronary artery bypass graft (CABG) surgery at Dunedin Hospital, New Zealand. Patients were excluded based on diagnosis of pre-operative AF or a left ventricular ejection fraction lower than 40%. Excised RAA samples were collected immediately in ice-cold Krebs-Henseleit buffer (KHB). Butanedione monoxime (BDM, 25 mM, Sigma) was included to arrest contraction and prevent cutting injury.

1. Human atrial trabeculae dissection and inotropy experiment

Samples of human right atrial appendages were transferred to the laboratory within 5 min of collection. Thin, linear, non-branching trabeculae were dissected from the samples using a stereomicroscope and mounted horizontally in a custom-built superfusion organ bath. Trabeculae were superfused with 37 °C 1.5 mM Ca^2+^ KHB (118.5 mM NaCl, 4.5 mM KCl, 1.0 mM MgCl_2_·6H_2_O, 0.33 mM NaH_2_PO_4_, 25 mM NaHCO_3_, 11 mM glucose, and 0.5 mM CaCl_2_ perfused with carbogen [95% O_2_ + 5% CO_2_], pH 7.4) and field stimulated at 1 Hz (suprathreshold voltage, 5 ms duration). Contracting trabeculae were stabilised for 1 h. During this time, the trabeculae were stretched incrementally using a micromanipulator until an increase in muscle length was not accompanied by an increase in developed force. Following equilibration, baseline isometric force of the trabeculae was determined from the steady-state contractions. Baseline spontaneous contraction propensity was assessed by 1-min cessation of external stimulus, as previously described (5). Trabeculae which developed spontaneous contractions (≥1 contraction) during the rest interval were categorised as spontaneously active. Following resumption of stimulation and re-stabilization, C18:1AC was added to the KHB solution at 25 µM and was superfused over each trabecula for 1 h. Trabeculae developed force and spontaneous contraction propensity was then re-assessed. Afterwards, the long-chain AC was washed out for 30 min and developed force as well as spontaneous contractions were assessed again. Data were analysed using LabChart 8.0 (ADInstruments). Trabeculae force output was normalised to muscle cross sectional area as determined by width x thickness x π.

1. Action potential measurements in human EHT models

Action potentials (APs) were measured by sharp microelectrode. For AP measurements, EHTs (after >30 days of culture) were placed in a solution containing 127 mM NaCl, 4.5 mM KCl, 1.5 mM MgCl_2_, 1.8 mM CaCl_2_, 10 mM Glucose, 22 mM NaHCO_3_, and 0.42 mM NaHPO_4_ equilibrated with O_2_-CO_2_ [95:5] at 36.5 ± 0.5 °C, pH 7.4. APs were continuously measured during baseline and perfusion with 25 µM C18:1AC for 20 min each. Electrical stimulation was performed for vEHT and aEHT at 1 Hz and 3.5 Hz, respectively. In 63% of experiments, APs were measured from the same impalement before and after C18:1AC application. In the remaining experiments, the signal was temporarily lost, but could be recovered at the same position. APs were analysed using the Lab-Chart software (ADInstruments).

1. Experiments on isolated cardiac myocytes

Ca^2+^ experiments in single cardiomyocytes were performed as described previously (6-8). Isolated adult ventricular myocytes were field-stimulated at 37 °C with a stimulation rate of 1.0 Hz and superfused with Tyrode’s solution containing: 130 mM NaCl, 5 mM KCl, 1 mM MgCl_2_, 1 mM CaCl_2_, and 10 mM Na-HEPES, pH 7.4. After 120 s, cardiac myocytes were superfused with Tyrode’s solution containing 1 to 25 µM of C18:1AC. Cytosolic Ca^2+^ and mitochondrial membrane potential (Δψ_m_) of intact cardiac myocytes were monitored by loading the cells with the fluorescent Ca^2+^ indicator indo-1 acetoxymethyl ester (AM, excitation: 𝜆=340  nm; emission 𝜆=405/485 nm, Thermo Fisher Scientific) and the potentiometric dye tetramethylrhodamine methyl ester (TMRM, excitation: 𝜆=540 nm; emission 𝜆=605 nm, Thermo Fisher Scientific), a membrane-permeant cation which accumulates across the inner mitochondrial membrane depending on Δψ_m_. Upon hypercontraction, cells were considered as dead. To determine SR Ca^2+^ load, 10 mM caffeine were directly applied to a single myocyte to provoke RyR2 opening and SR Ca^2+^ release. To calculate the arrhythmic score, number of contractions and stimulations were counted and the number of contractions was divided by the numbers of stimulations. A cell with an arrhythmic score above 1 was considered as arrhythmic and below 1 as bradycardic. To calculate the arrhythmic score in unstimulated iPSC, the time interval between each of 21 Ca^2+^ transients was measured before and after treatment with C18:1AC or short-chain AC. Its variation was used as an arrhythmia index.

1. Protein detection

Proteins were detected by standard Western blot technique. Antibodies were directed against sarcoplasmic-endoplasmic reticulum calcium ATPase 2a (SERCA2A, Invitrogen, #MA3-919, 1:1000 in 1% milk), phospholamban (PLN, Novus Biologicals, #NBP2-19807, 1:1000 in 1% milk), Ca^2+^/calmodulin-dependent protein kinase II (CaMKII, Becton-Dickinson, #611292, 1:1000 in 5% bovine serum albumin), phospholamban serin 16 phosphorylation (pPLN, Badrilla, #A010-12, 1:2000 in 1% milk), protein kinase A substrate phosphorylation (PKAsub, Cell Signaling Technologies, #9621, 1:1000 in 5% bovine serum albumin), CaMKII threonine 286 phosphorylation (pCaMKII, Cell Signaling Technologies, #12716, 1:1000 in 5% bovine serum albumin) and CaMKII methionine 281/282 oxidation (o-CaMKII, Merck, #07-1387, 1:1000 in 5% bovine serum albumin). For oxidized CaMKII Western blot, cells were harvested and lysed under non-reducing conditions in a maleimide containing buffer. For all other blots, β-mercaptoethanol containing reducing buffer was used. Proteins were separated on either 10% (all CaMKII blots, PKAsub) or 15% (all others) acrylamide containing SDS-PAGE gels and blotted onto either nitrocellulose (all CaMKII blots, PKAsub) or PVDF membranes (all others). Blots were blocked in either 10% milk (for all antibody incubation reactions in milk) or 5% bovine serum albumin (for all antibody incubation reactions in bovine serum albumin). Blots were blocked for 1 h, incubated at 4 °C over night, subsequently incubated with corresponding horseradish peroxidase coupled secondary antibodies for 1 h and visualized using ECL substrate (Pierce). Western blots were analyzed using ImageLab software (BioRad).

1. Mitochondrial respiration and determination of ΔΨ_m_ in isolated mitochondria and atrial EHT

An Oxygraph-2K instrument (Oroboros) was used for respirometric analysis. Respiration of isolated mitochondria was measured at 37 °C in respiration buffer, containing (in mM) KCl 137, KH_2_PO_4_ 2, EGTA 0.5, MgCl_2_ 2.5, HEPES 20, at pH 7.2 with stirring at 750 rpm, while respiration from intact EHT was measured in MiR05 buffer, containing (in mM) EGTA 0.5, MgCl_2_ 3, K-lactobionate 60, taurine 20, KH_2_PO_4_ 10, HEPES 20, sucrose 110, 1 g/L fatty acid free BSA, adjusted to pH 7.1 with KOH at 25 °C with 540 rpm stirring. Respiratory state 2 was induced by adding either pyruvate and malate or glutamate and malate (5 mM each) or all three of them for intact EHT (10 mM glutamate). Maximal oxygen consumption coupled to ADP phosphorylation (state 3) was stimulated by adding saturating ADP (1 mM for isolated mitochondria, 5 mM for EHT). To additionally probe complex II respiration, succinate was then added for EHT measurements. Subsequently, in isolated mitochondria state 4 was obtained by inhibiting complex V of the respiratory chain with oligomycin (1.2 µM). Finally, the protonophore 2,4-dinitirophenol was titrated (25 µM steps, 125 µM steps in EHT) to induce maximal non-coupled respiration. In isolated murine cardiac mitochondria from left and right ventricle, the effect of C18:1AC (12.5 and 25 µM) on ADP-stimulated respiration (1 mM) was investigated using either glutamate and malate (G/M) or pyruvate and malate (P/M), 5 mM each, as substrates. In human atrial mitochondria from patients in sinus rhythm, P/M was used as substrate and respiration was analysed using successive addition of C18:1AC (5 µM steps). Respiration on human atrial mitochondria from the Würzburg cohort was measured using the following substrates: 1 mM carnitine, 3 mM malate, 10 µM palmitoyl-CoA and 10 µM C18:1AC, followed by increasing ADP concentrations (0.03, 0.1, 0.3 and 1 mM), 1.25 µM oligomycin and 10 µM dinitrophenol (DNP) in the presence of bovine serum albumin (BSA, 1 mg/mL). For respiration measurements from atrial EHT, tissues were permeabilized with 0.005% (w/v) of digitonin and 2 EHTs were pooled for each measurement. ΔΨm was assessed using tetramethylrhodamine methyl ester (TMRM, 1 µM), which accumulates in the mitochondrial matrix upon polarization of the inner mitochondrial membrane, leading to fluorescence quenching. TMRM fluorescence was recorded using an Oroboros O2k Smart Fluo-Sensor Green, excitation LED 525 nM. Oxygraph plus and DatLab (Oroboros) were used for data acquisition and analysis.

1. Preparation of EHT for mass spectrometry for C18:1AC detection

To detect C18:1AC in whole human EHT (cardiomyocytes within fibrin matrix), the tissues were homogenised in ice-cold acetonitrile using a Tissue Lyser disruption device (QIAGEN), centrifuged for 15 min (9300 g, 4 °C), and the supernatant was used for high pressure liquid chromatography electrospray ionization tandem mass spectroscopy (HPLC-ESI-MS/MS). For matrix digestion and dissociation into single cardiomyocytes, EHTs were washed with 1x Earl’s Balanced Salt Solution (EBSS), and incubated in a papain solution (10 U/mL papain (Sigma), 1 mM EDTA, and 5.5 mM L-cysteine-HCl in 1x EBSS) at 37 °C for 20-40 min. Following centrifugation for 10 min at 100 g, cells were resuspended in (i) ice-cold acetonitrile for subsequent lysis (as described above) or (ii) in PBS for additional subcellular fractionation. Cell fractionation was performed with the Cell Fractionation kit (Cell Signaling) according to the manufacturer’s instructions. For one fractionation, dissociated hiPSC-derived cardiomyocytes from three EHTs were pooled to achieve the minimum number of cells needed according to the protocol (~2.5 Mio cells). All prepared samples were stored at -80 °C before HPLC-ESI-MS/MS measurements. Prior to HPLC-ESI-MS/MS measurements, freeze-drying was performed for all samples (except for the subcellular fractionation samples) in open vessels at a pressure of 0.1 mbar using the Lyovac GT 2 Amsco/FinnAqua System (GEA Pharma Systems). The residues were dissolved in 70% acetonitrile/30% water.

1. HPLC-ESI-MS/MS measurements

C18:1AC concentrations in all samples were quantified by HPLC-ESI-MS/MS using an Agilent 1100/1200 Series HPLC system (Agilent 1200 Degasser (G1379B), Agilent 1200 Binary Pump (G1312A), Agilent 1100 ALS (G1329A), Agilent 1200 Col Comp (G1316A), Agilent 1100 Controller (G1323B)) coupled to a Sciex API 3200 triple quadrupole mass spectrometer. All data were recorded and analysed using Analyst software version 1.6.2 (Sciex). The separation was carried out on a 50 x 2.1 mm, 3 µm Merck Purospher Star HIBar HR endcapped C8 column using a mobile phase of methanol containing 10% acetonitrile (solvent A) and water containing 1.0% formic acid (solvent B). All solvents were of HPLC-MS grade. The gradient used was as follows: 1 min isocratic 40% of solvent A were linearly raised for 3 min to 100% solvent A followed by further 4 min of isocratic 100% solvent A. Afterwards the column was flushed back to starting conditions and equilibrated for another 4 min. The column was tempered at 40 °C and the flow rate was 300 µL/min. All measurements were carried out using positive ionization and in multiple reaction monitoring (MRM) mode. A Turbo V Source was used for the ionization of samples using zero grade air as nebulizer and auxiliary gas, while nitrogen was used as curtain gas and collision gas. Source parameters were: curtain gas 20 psi, nebulizer gas 35 psi, auxiliary gas 45 psi, collision gas 10·10-5 torr, ion spray voltage 5500 V and source temperature 350 °C. MRM transitions were as follows: m/z 426 🡪 85 was used as a quantifier (declustering potential (DP): 51 V, entrance potential (EP): 9 V, cell entrance potential (CEP): 26 V, collision energy (CE): 41 V, collision cell exit potential (CEP): 4 V) and m/z 426 🡪 60 (DP: 51 V, EP: 9 V, CEP: 26 V, CE: 45 V, CXP: 0 V) as well as m/z 426 🡪 55 were (DP: 51 V. EP: 9 V, CEP: 26 V, CE: 81 V, CXP: 4 V) were used as qualifiers. For each SRM a dwell time of 50 ms was used. Calibration was carried out as external calibration with C18:1AC solutions in 10% acetonitrile in a range between 5 ng/mL and 200 ng/mL. Samples were diluted with 10% acetonitrile when necessary to obtain a concentration within calibration range.

1. Human cohorts

Würzburg cohort

The use of myocardial tissue for this study was approved by the local ethics board (ethics approval no. 143/17-sc). Samples from patients in sinus rhythm (n=29), with paroxysmal AF (PAF, n=10) and with sustained AF (SAF, n=6) were collected. Definition of PAF (lasting less than 2 days) and sustained AF (lasting more than 7 days) was in accordance with the European Society of Cardiology (ESC) classification (9). Myocardial tissue samples from the left atrial appendage were collected during cardiac surgery at the University Clinic Würzburg, Germany. Excised samples were immediately stored in Custodiol tissue storage solution (Köhler) and cleared from debris. Mitochondria isolation was performed as described previously.(6, 10)

New Zealand cohort

The cohort comprised patients with coronary artery disease who underwent coronary artery bypass graft (CABG) surgery at Dunedin Hospital, New Zealand. The use of serum samples for this study was approved by the local ethics board (ethics approval no. #LRS12-01-001AM1-AM17) and in line with the Declaration of Helsinki. Patients requiring emergency CABG and those undergoing concomitant cardiac surgical procedures in addition to CABG were excluded. Serum samples from patients in sinus rhythm (n=28), with PAF (n=13), and with SAF (n=11) were used for metabolome analysis (section 16).

BiomarCaRE

For BiomarCaRE, a case-cohort design was applied including different European cohorts: FINRISK97 (Finland), Moli-sani (Italy), Danish-Multinational Monitoring of Trends and Determinants in Cardiovascular Disease (DanMONICA, Denmark), and Scottish Heart Health Extended Cohort (SHHEC, United Kingdom). The studies were approved by the respective local ethics boards and all participants provided written informed consent (FINRISK97: 82/2001, Moli-sani: Prot.pdc.P.99/A.931/03-128-04/C:E./2004, DanMONICA: 1980-272-2 1051NE/bt 2-16-2/43(85) KA 90238). All participating cohort studies complied with the Declaration of Helsinki. The cohorts were based on random samples of the general adult population. Classic cardiovascular risk factors of the participants were assessed and blood samples were taken at baseline, and the cohorts were prospectively followed up for incident AF. Data from the cohorts were harmonised in the MORGAM and BiomarCaRE projects (11). The BiomarCaRE case-cohort set consists of a weighted random subsample of those individuals of the original cohort (>70,000 individuals) who had no history of myocardial infarction, stroke, heart failure, and AF and who were selected independently of the definition of cases, plus all individuals of the original cohort who developed AF during the follow-up period (12). Overall, baseline serum samples of 9,028 individuals with 1,437 incident AF events were analysed.

1. Metabolome analysis in human serum samples

By mass spectrometry, 186 metabolites from 6 different compound classes were analysed using the AbsoluteIDQ p180 kit (Biocrates). The metabolome measurements were performed with 10 µL of serum from each sample according to the manufacturer's instructions. The MetIDQ software (Biocrates) was used for automated data analysis. In BiomarCaRE, internal standards for appropriate identification and quantification were used as described previously (12). In the New Zealand cohort, three replicates of a pooled plasma sample obtained from healthy volunteers were added to each plate for normalization.

1. Data processing and statistical analysis of metabolome data

Normalised metabolites were excluded if >30% of measurements were missing or >50% of measurements were below the level of detection. Samples were excluded when >50% of metabolites were missing (12). For final analysis, 45 metabolites were excluded after quality controls. In BiomarCaRE, Multivariate Imputations by Chained Equations (MICE) was used to fill missing values. Twenty imputed datasets were generated. A log-normal model was used to impute missing metabolite concentrations. In particular, metabolite imputed values were drawn from a truncated log-normal distribution, if the missing values were known to be below the limit of detection. For other variables, predictive mean matching was used to impute missing values (Table S3). Both the event indicator (AF) and the Nelson-Aalen estimate of the cumulative survival function of the time to AF were included in the imputation model. The imputation was performed separately for each combination of sex and study centre (FINRISK97, Moli-sani, DanMONICA, and SHHEC). Associations with time-to-AF were assessed for each of the 141 log-transformed metabolite concentration values individually by applying weighted Cox proportional hazard models adjusted for body mass index (BMI), systolic blood pressure (SBP), antihypertensive treatment, diabetes, total cholesterol, sex, daily smoking, study centre, and age at baseline examination. Adjustment for age was achieved using this variable as the time scale. Adjustment for sex and study centre was performed by using these variables as stratification variables in the Cox models. The case-cohort design was taken into account in the Cox regressions using methods as described by Kulathinal et al. (13). Individuals were weighted with inverse subcohort sampling probability weights and robust standard errors were used. After Bonferroni correction for multiple testing, significance was met if p-values were <4.55 x 10^-4^ (0.05/110). The methods described in Gao et al (14) were used to estimate the number of tests to use in the Bonferroni correction (n=110), namely the number of effective tests, which is calculated as the number of principal components of the log-metabolites that explain 99.5% of the variation. Hazard ratios for the log-metabolites were reported per 1 standard deviation (SD) increase. The SD was calculated in the subcohorts. Cox regression analyses for each study centre were also performed. The Kaplan-Meier method was used to estimate survival curves. The methods described by Kulathinal et al. (13) were used to account for the case-cohort design. Statistical methods were implemented in the R statistical software version 3.5.1 (15).

For the New Zealand cohort, the following quality control and data processing steps were performed. For each plate the measured concentrations of the metabolites were divided by the median concentration of the pooled quality control samples. Subsequently, for each metabolite the median of the plate-specific medians of the pooled samples was calculated to reset the concentration to the original scale (µM concentrations). For each metabolite, the plate-specific coefficient of variation (CV) was calculated based on the pooled samples. Furthermore, based on the duplicate measurements, Pearson’s correlation coefficients were calculated. Only metabolites with 1) at least a CV <25% on two plates or 2) a Pearson’s correlation coefficient >0.5 or acceptable visual control of agreement were included in the final data sets, resulting in 163 metabolites used for subsequent analysis. For the statistical analysis, metabolite concentrations per sample were derived as the mean of the two technical replicates independent of the level of detection (LOD). Differential expression of long-chain ACs was assessed using the following linear regression model: Metabolite concentration ~ β_0_ + β_1_ ⋅ AF subtypes + β_2_ ⋅ age + β_3_ ⋅ sex + β_4_ ⋅ BMI + β_5_ ⋅ systolic blood pressure + β_6_ ⋅ diastolic blood pressure + β_7_ ⋅ diabetes + β_8_ ⋅ ever smoker + β_9_ ⋅ ejection fraction + ε. Two-sided t-statistics for β1 was evaluated to determine persistent AF association. False discovery rate (FDR) was computed based on the Benjamini-Hochberg procedure including all 12 quantified long-chain ACs (C14, C14:1, C14-OH, C14:2, C14:2-OH, C16, C16:1, C16:1-OH, C16-OH, C18, C18:1, C18:2). Additionally, the same linear regression model was applied using log2-transformed concentrations on all quantified metabolites (163, no imputation) including FDR on all metabolites.

**Additional file 1: Tables and figures**

**Additional file 1: Table S1. Baseline characteristics of the Würzburg patient cohort.**

Baseline characteristics are given as quartiles for continuous variables (median, 25^th^ percentile, 75^th^ percentile) and as percentage for binary variables. PAF=paroxysmal atrial fibrillation, SAF=sustained atrial fibrillation.

| **Variable** | **No AF** (n=28) | **PAF** (n=9) | **SAF** (n=6) |
| --- | --- | --- | --- |
| Examination age (years) | 67.9  (58.4-76.6) | 75.6  (67.1-79.1) | 75.7  (70.3-80.5) |
| Females % | 21.4 | 11.1 | 33.3 |
| BMI (kg/m²) | 28.4  (25.2-31.1) | 27.6  (25.8-29.3) | 26.9  (25.0-27.9) |
| Diabetes % | 25.0 | 55.6 | 16.7 |
| Ejection fraction in % | 55.00  (53.0-61.5) | 54.00  (54.0-65.6) | 51.00  (45.5-59.5) |

**Additional file 1: Table S2. Baseline characteristics of the New Zealand patient cohort.**

Baseline characteristics are given as quartiles for continuous variables (median, 25^th^ percentile, 75^th^ percentile) and as percentage for binary variables. PAF=paroxysmal atrial fibrillation, SAF= sustained atrial fibrillation.

| **Variable** | **No AF** (n=28) | **PAF** (n=13) | **SAF** (n=11) |
| --- | --- | --- | --- |
| Examination age (years) | 69.5  (66.8-72.3) | 71.0  (63.0-79.0) | 76.0  (73.0-78.0) |
| Females % | 21.5 | 38.5 | 9.0 |
| BMI (kg/m²) | 29.3  (25.6-33.8) | 31.4  (27.0-35.5) | 28.1  (27.3-29.3) |
| Systolic BP (mmHg) | 134.5  (127.7-145.0) | 134.0  (119.0-138.0) | 130.0  (105.0-134.5) |
| Diastolic BP (mmHg) | 78.0  (70.0-85.0) | 77.0  (69.0-81.0) | 70.0  (65.0-74.0) |
| Ejection fraction % | 51.3  (47.5-58.0) | 57.5  (47.0-67.8) | 52.6  (37.8-58.1) |
| Ever smoker % | 53.6 | 53.9 | 63.6 |
| Diabetes % | 17.9 | 30.8 | 18.2 |

**Additional file 1: Table S3A. Baseline characteristics of the overall BiomarCaRE case-cohort study population.**

Baseline characteristics for all the representative subcohorts of the BiomarCaRE case cohort set given as quartiles for continuous variables (median, 25^th^ percentile, 75^th^ percentile) and as percentage for binary variables.

| **Variable** | **Overall** (n=9028) | **Women**  (n=3583) | **Men**  (n=5445) |
| --- | --- | --- | --- |
| Examination age (years) | 56.7  (49.6-61.7) | 58.5  (50.5-62.3) | 55.6  (48.1-61.4) |
| BMI (kg/m²) | 26.1  (23.6-29.0) | 25.7  (22.8-29.2) | 26.3  (24.1-29.0) |
| Systolic BP (mmHg) | 134.5  (121.0-150.0) | 134.0  (119.0-150.0) | 135.0  (122.0-151.0) |
| Diastolic BP (mmHg) | 82.0  (74.0-89.0) | 80.0  (73.0-87.0) | 83.0  (76.0-90.0) |
| Total cholesterol (mg/dL) | 228.2  (201.1-259.1) | 235.9  (205.0-270.7) | 224.3  (197.2-255.0) |
| HDL cholesterol (mg/dL) | 54.0  (44.9-65.0) | 61.5  (51.4-72.7) | 49.9  (42.0-59.0) |
| LDL cholesterol (mg/dL) | 144.2  (119.6-172.8) | 147.6  (121.6-178.1) | 142.4  (118.7-169.4) |
| Triglycerides (mg/dL) | 119.0  (86.0-172.0) | 108.0  (80.0-151.0) | 126.0  (90.0-189.0) |
| Daily smoker % | 31.4 | 28.9 | 33.1 |
| Diabetes % | 4.6 | 3.6 | 5.3 |
| Hypertension % | 48.1 | 46.1 | 49.5 |
| Antihypertensive % | 17.1 | 19.2 | 15.8 |
| Cholesterol lowering medication % | 6.2 | 7.2 | 5.6 |
| Incident atrial fibrillation % | 15.9 | 16.5 | 15.5 |

**Additional file 1: Table S3B. Baseline characteristics of the subcohorts comprising the BiomarCaRE case-cohort study population.**

Baseline characteristics for all the representative subcohorts of the BiomarCaRE case cohort set given as quartiles for continuous variables (median (25^th^ percentile, 75^th^ percentile)) and as absolute numbers (percentage in brackets) for binary variables. *=data not available

| **Variable** | **DanMONICA** (N=3378) | **FINRISK97** (N=1741) | **Moli-sani** (N=1496) | **SHHEC** (N=2413) |
| --- | --- | --- | --- | --- |
| Examination age (years) | 50.9  (41.1-60.7) | 59.0  (50.4-65.4) | 67.5  (57.6-74.8) | 53.5  (47.8-58.5) |
| Male (%) | 1941 (57.5) | 1110 (63.8) | 910 (60.8) | 1484 (61.5) |
| BMI (kg/m²) | 24.9  (22.7-27.7) | 27.2  (24.6-29.9) | 28.3  (25.7-31.6) | 25.7  (23.4-28.4) |
| Systolic BP (mmHg) | 126.0  (115.0-139.0) | 142.0  (129.0-157.0) | 148.0  (135.0-164.0) | 133.0  (121.0-149.0) |
| Diastolic BP (mmHg) | 80.0  (72.0-87.0) | 85.0  (78.0-92.0) | 82.5  (76.0-89.0) | 83.0  (75.0-90.0) |
| Total cholesterol (mg/dL) | 232.0  (201.1-263.0) | 220.4  (193.4-247.5) | 211.0  (184.0-239.0) | 243.6  (216.6-278.4) |
| HDL cholesterol (mg/dL) | 54.9  (45.3-66.9) | 51.0  (42.9-60.7) | 54.0  (45.0-65.0) | 54.5  (45.2-65.7) |
| LDL cholesterol (mg/dL) | 148.6  (122.8-178.2) | 139.2  (117.1-164.2) | 128.7  (105.0-152.8) | 154.1  (127.1-184.2) |
| Triglycerides (mg/dL) | 104.0  (78.0-145.0) | 117.0  (87.0-166.0) | 117.0  (85.0-159.0) | 150.0  (106.0-227.0) |
| Daily smoker (%) | 1430 (42.3) | 341 (19.6) | 216 (14.4) | 851 (35.3) |
| Diabetes (%) | 92 (2.7) | 121 (7.0) | 165 (11.0) | 40 (1.7) |
| Hypertension (%) | 1019 (30.2) | 1093 (62.8) | 1156 (77.3) | 1078 (44.7) |
| Antihypertensive  (%) | 306 (9.1) | 329 (18.9) | 699 (46.7) | 210 (8.7) |
| Cholesterol lowering medication (%) | 5 (0.6) | 64 (4.6) | 158 (11.2) | * |
| Incident atrial fibrillation (%) | 523 (15.5) | 237 (13.6) | 317 (21.2) | 360 (14.9) |

**Additional file 1: Table S7. Number of individuals included in the BiomarCaRE case-cohort set.**

|  | **Overall** | **DanMONICA** | **FINRISK97** | **Moli-sani** | **SHHEC** |
| --- | --- | --- | --- | --- | --- |
| All | 9028 | 3378 | 1741 | 1496 | 2413 |
| No AF during FU | 7591 | 2855 | 1504 | 1179 | 2053 |
| AF during FU | 1437 | 523 | 237 | 317 | 360 |


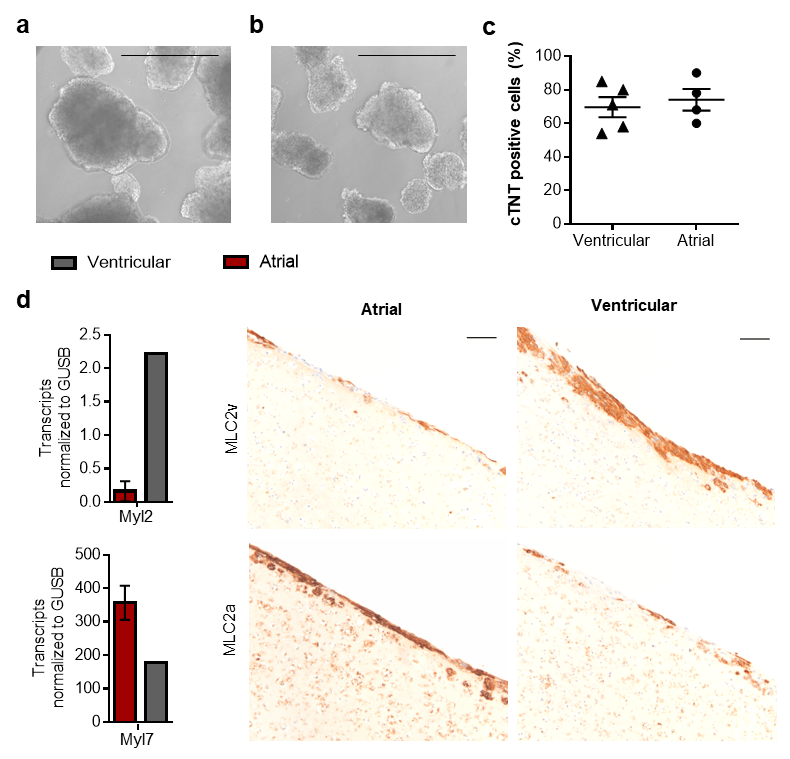
 **Additional file 1: Fig. S1. Differentiation of human induced pluripotent stem cells into ventricular and atrial cardiomyocytes.** Embryoid bodies after induction of **(a)** ventricular and **(b)** atrial cell fate on day 16 of differentiation (scale bar=600 µm). **(c)**Proportion of troponin T (cTNT) positive cells at the end of each independent ventricular and atrial differentiation run (day 17), as determined by flow cytometry**. (d)** Normalized transcript abundance of the ventricular and atrial isoforms of the myosin regulatory light chain (MYL2 and MYL7) and paraffin sections of aEHT and vEHT stained for MLC2v and MLC2a. Transcript abundance was normalized to GUSB transcript abundance and quantified in hiPSC-derived cardiomyocytes at the end of ventricular (n=1) and atrial (n=2-3) differentiation runs on day 17. Histological analyses were performed in 37 day-old aEHT and vEHT (scale bar=100 µm).


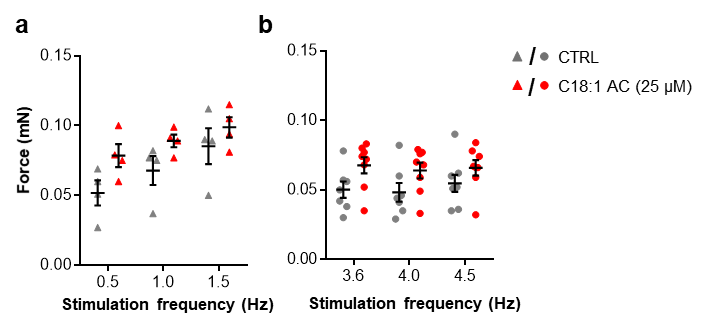


**Additional file 1: Fig. S2. Force-frequency relationship.** Contractile force of both EHT models after 20 min incubation with C18:1AC (25 µM) or solvent only (control, CTRL) at increasing pacing frequency. Due to their different spontaneous beating frequency, different stimulation frequencies were applied for **(a)** vEHT (0.5-1.5 Hz, 0.8 mM calcium, n=4/1 per group) and **(b)** aEHT (3.6-4.5 Hz, 1.4-1.5 mM calcium; n=8/1 per group, 2 independent experiments). Mean ± SEM. Numbers given as total number of EHTs/number of batches.


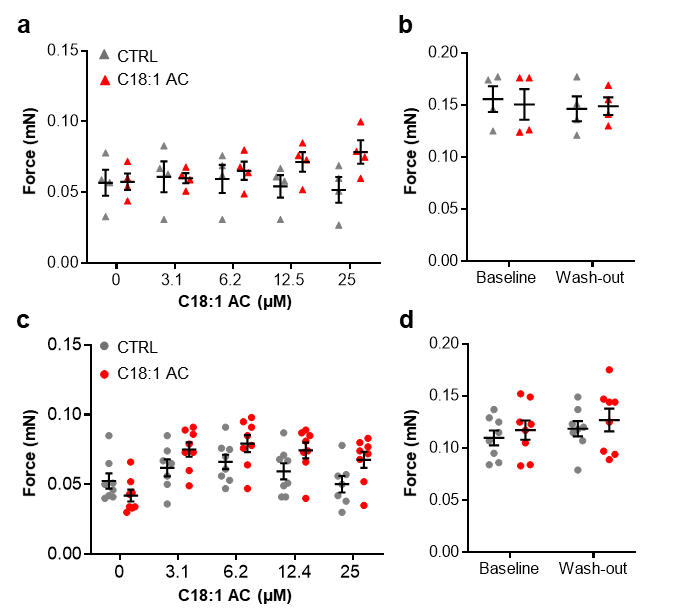


**Additional file 1: Fig. S3. Short-term effect of C18:1AC on contractility of human EHT.** Raw data of force of **(a)** vEHT (0.5 Hz, 0.8 mM calcium, n=4/1 per group) and **(c)** aEHT (3.6 Hz, 1.4-1.5 mM calcium; n=8/1 per group, 2 independent experiments) exposed to increasing concentrations of C18:1AC under electrical stimulation. Inotropic effect compared to time-matched controls exposed to solvent only (vEHT n=4 per group; aEHT n=8 per group, two-way ANOVA plus Bonferroni’s post-test for multiple comparisons). (**b and d**) Force, one day after exposure and wash-out. No difference between controls and C18:1AC exposed vEHT or aEHT, measured without electrical stimulation. Mean ± SEM. Numbers given as total number of EHTs/number of batches.


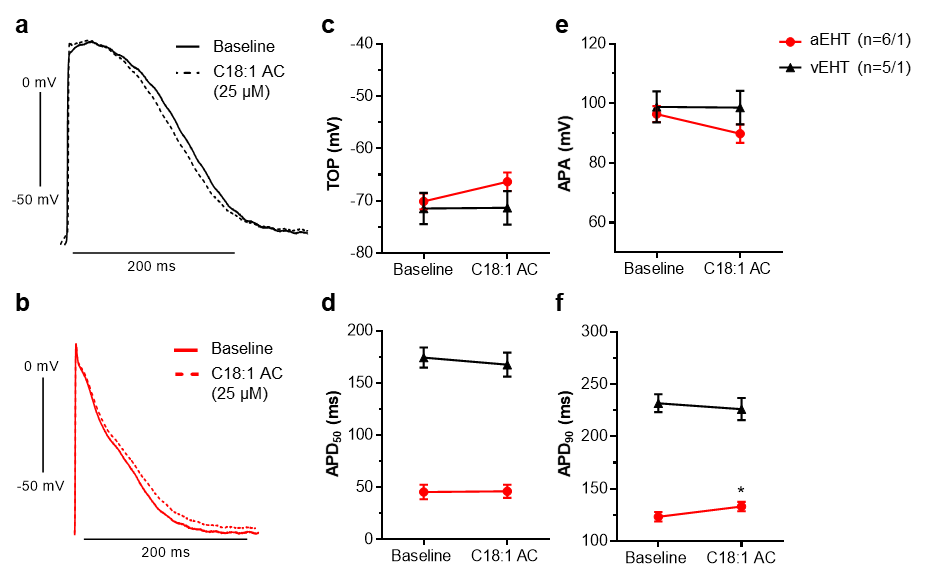


**Additional file 1: Fig. S4. Action potentials of C18:1AC exposed human vEHT and aEHT.** Original traces of action potentials (APs) of human **(a)** vEHT and **(b)** aEHT before (solid line) and after 20 min of exposure to C18:1AC (25 µM, dotted line). Mean values ± SEM of the **(c)** take-off potential (TOP), **(d)** AP duration at 50% repolarization (APD_50_), **(e)** AP amplitude (APA), and **(f)** AP duration at 90% repolarization (APD_90_) before and after 20 min of C18:1AC exposure. Paired t-test, *p<0.05. AP recordings performed at 37 °C. Stimulation rate 1 Hz for vEHT and 3.5 Hz for aEHT. Numbers given as total number of EHTs/number of batches.


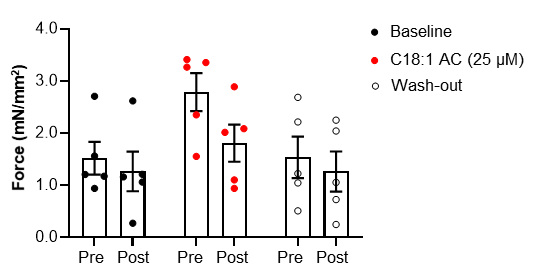


**Additional file 1: Fig. S5. Post-pause force of atrial trabeculae after C18:1AC exposure.** Force of atrial trabeculae before (pre) and after a 1-min pause of electrical stimulation (post) at baseline, after 60 min of exposure to 25 µM C18:1AC, and after a wash-out of 30 min. Two-way ANOVA plus Bonferroni’s post-test for multiple comparisons, n=5/5 (number of trabeculae/number of patients). Mean ± SEM.


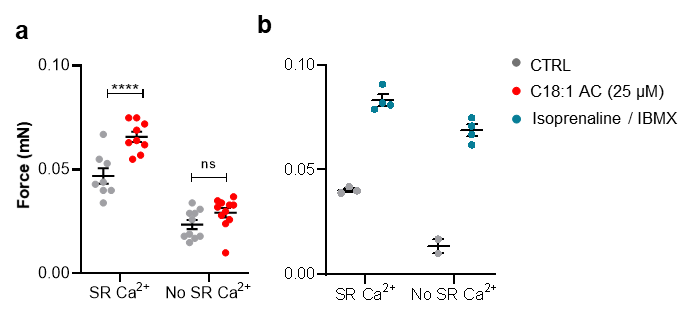


**Additional file 1: Fig. S6. Positive inotropic effect of C18:1AC. (a)** Raw data of force of human aEHT (3.6 Hz, n=8-12/2 per group, three independent experiments) after exposure to C18:1AC (25 µM) under electrical pacing in the presence of SR Ca^2+^ and without. SR Ca^2+^ was reduced by pre-incubating aEHT with ryanodine (1 µM) and cyclopiazonic acid (10 µM) for 20 min. Two-way ANOVA plus Bonferroni’s post-test for multiple comparisons (****p<0.0001, n=8‑12, 1.2-1.5 mM Ca^2+^, three independent experiments). **(b)** The combination of the β-adrenergic activator isoprenaline (300 nM) and the phosphodiesterase inhibitor IBMX (10 µM) was used as positive control for aEHT (3.6 Hz, n=2-5/1) which induced a positive inotropic effect in the presence of SR Ca^2+^ and without (n=2-4, 1.3 mM Ca^2+^). Mean ± SEM. Numbers given as number of EHTs/number of batches. IBMX=isobutylmethylxanthine, SR=sarcoplasmic reticulum.

**
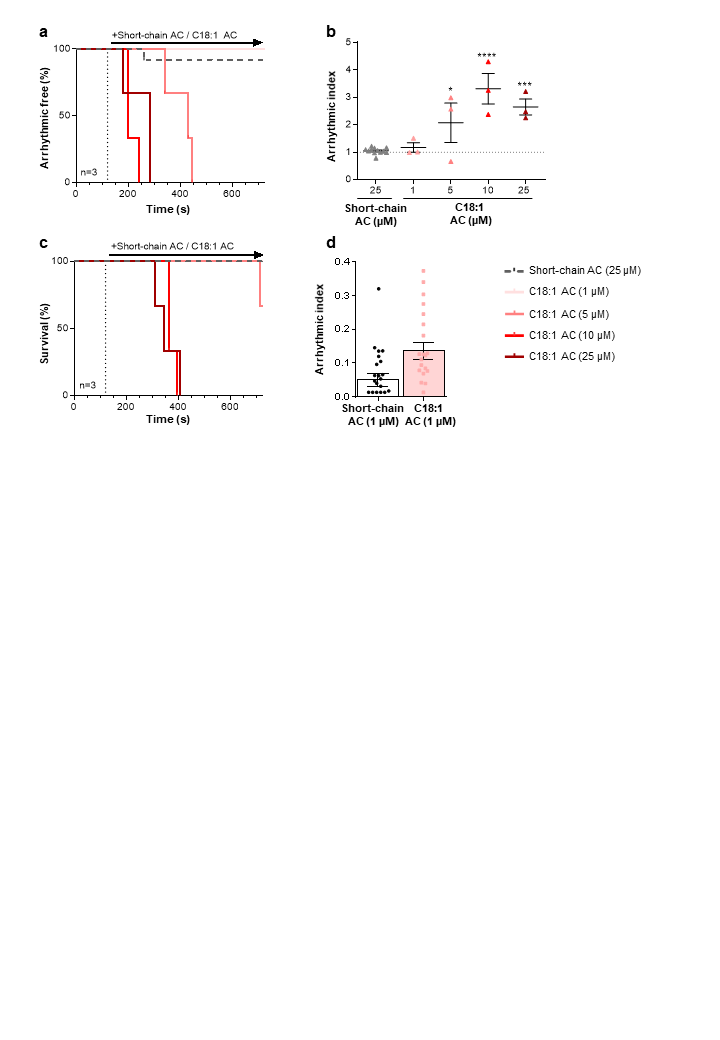
**

**Additional file 1: Fig. S7. Arrhythmogenic potential of C18:1AC.** **(a and b)** C18:1AC induced arrhythmias in murine ventricular cardiomyocytes at concentrations higher than 1 µM, defined as additional beats during electrical stimulation (1 Hz, mean ± SEM). Exposure to the short-chain AC had no effect (n=3-12/3-4 mice per group). **(c)** After 120 s of baseline measurement, isolated murine ventricular cardiomyocytes were exposed to different concentrations of indicated ACs (1-25 µM). Cellular mortality rate increased concentration-dependently at concentrations beyond 10 µM. The short-chain AC had no effect (n=2-12/3-4 mice per group). **(d)** Unstimulated ventricular iPSC-CM were superfused for 120 s with Tyrode’s solution and then superfused with additionally either C18:1AC or short-chain AC for 420 s. At the end of the protocol the time interval between two Ca^2+^ transients was quantified. Its variation was used as an arrhythmia index. One-way ANOVA plus Dunnett’s post-test for multiple comparisons vs. controls exposed to short-chain AC, *p<0.05, ***p<0.001, ****p<0.0001.


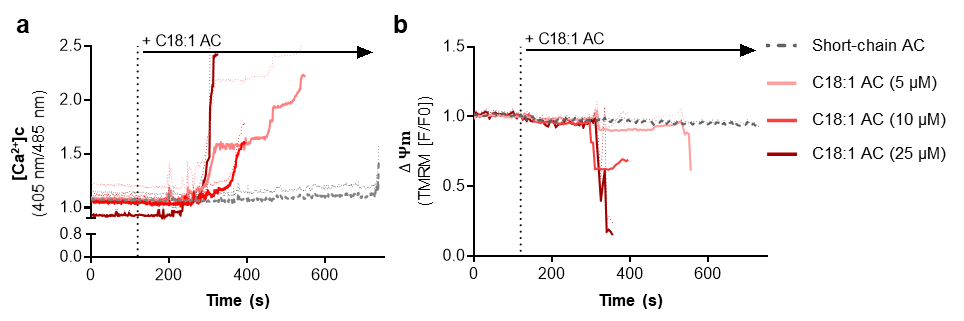


**Supplemental Fig. S8. Influence of C18:1AC on calcium load and mitochondrial membrane potential in murine ventricular cardiomyocytes.** Cardiomyocytes were loaded with the calcium-dye Indo-AM to determine cytosolic Ca^2+^ and the voltage dye TMRM (tetramethylrhodamine methyl ester) for assessment of mitochondrial membrane potential, ΔΨm. **(a)** Intracellular Ca^2+^ concentration was obtained by quantifying the 405/485 ratio for Indo-AM after adding C18:1AC (5-25 µM). **(b)** Mitochondrial membrane potential of cardiomyocytes exposed to C18:1AC depicted as TMRM fluorescence intensity normalized to baseline (F/F0, n=3-6 mice).

**
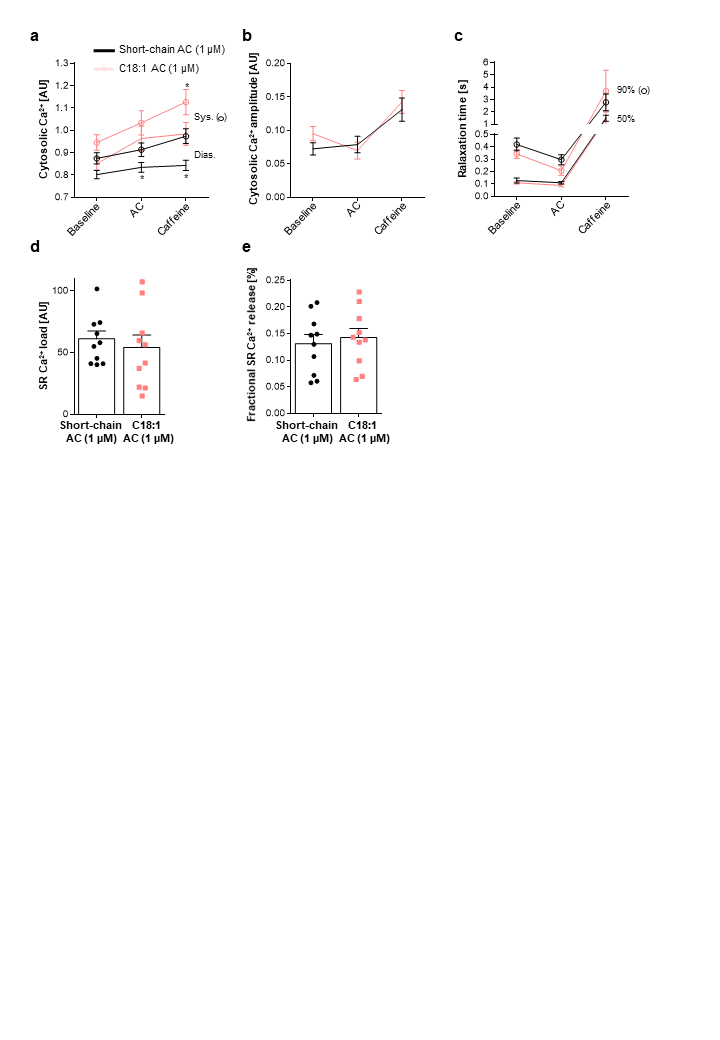
**

**Additional file 1: Fig. S9. Influence of C18:1AC on systolic and diastolic calcium load in murine ventricular cardiomyocytes.** Cardiomyocytes were loaded with the calcium-dye Indo-AM to determine cytosolic Ca^2+^ and were electrically stimulated at 1 Hz. After 120 s, C18:1AC or short-chain AC were washed in for 420 s and a pulse of 10 mM caffeine was applied to release SR Ca^2+^ and to determine SR-load. **(a)** Diastolic and systolic Ca^2+^ concentrations were monitored during the experiment. Diastolic Ca^2+^ concentration increased more during C18:1AC treatment compared to short-chain AC treatment. Ca^2+^ transient amplitude **(b),** Ca^2+^ decay kinetics **(c)**, SR load **(d)**, and fractional Ca^2+^ release **(e)** remained uneffected. Mean ± SEM. 10 cells from 2 mice per condition, Two-way ANOVA plus Bonferroni’s post-test for multiple comparisons vs. controls (a-c), unpaired t-test (d-e).

**
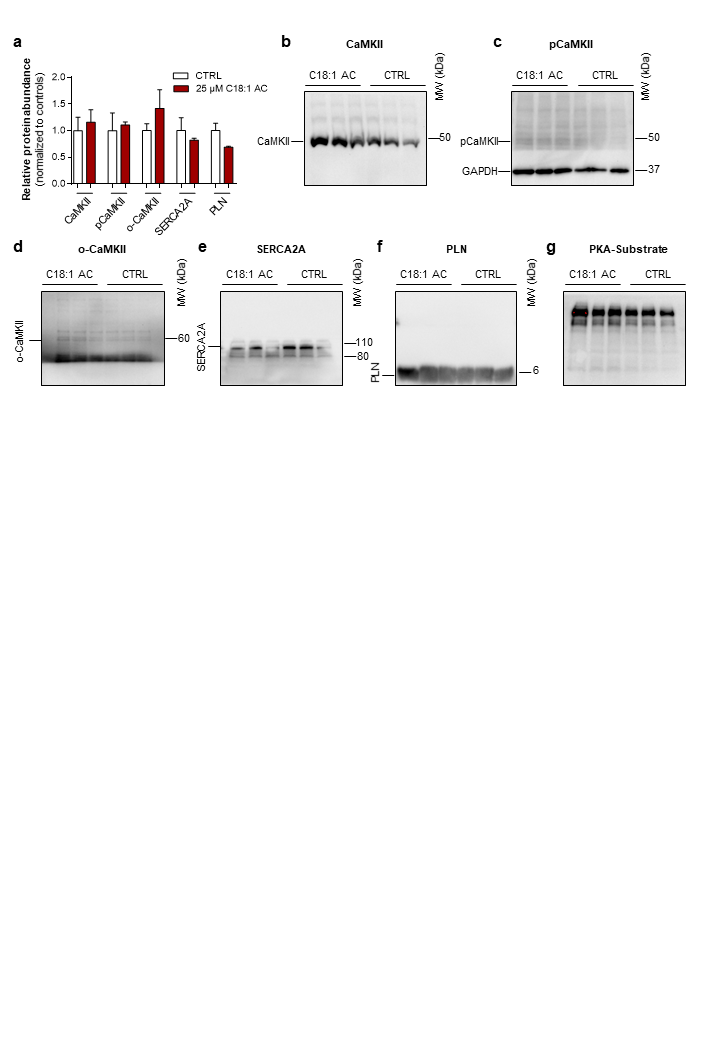
**

**Additional file 1: Fig. S10. Western blots of calcium handling related proteins. (a)** Quantification of protein abundance from following images. Human iPSC-derived cardiomyocytes were cultivated in standard 2D cell culture and treated with 25 µM of C18:1AC for 20 min. Band intensity was normalized to GAPDH band intensity and average of controls was set to one. Calcium ATPase 2a (SERCA2A), phospholamban (PLN), Ca^2+^/calmodulin-dependent protein kinase II (CaMKII), CaMKII threonine 286 phosphorylation (pCaMKII,) and CamKII methionine 281/282 oxidation (o-CaMKII) were probed. n=3 biological replicates per condition. No significant differences between conditions. **(b-f)** Original data for (a). **(g)** PKA-Substrate Western blot did not show obvious differences between groups.


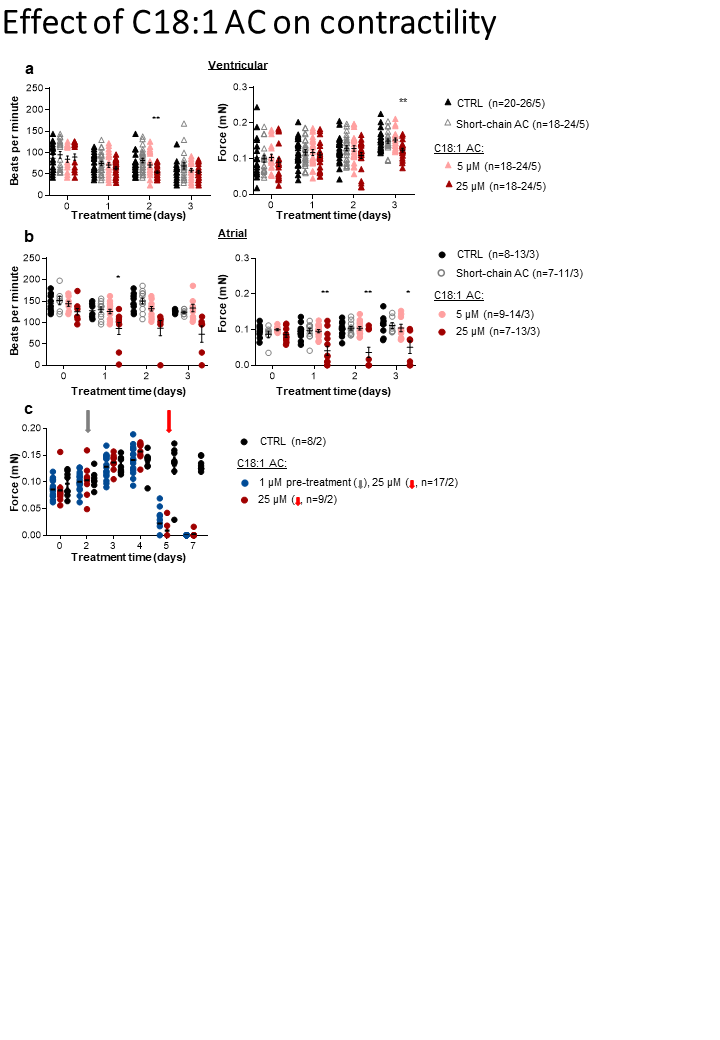


**Additional file 1: Fig. S11. Time and concentration-dependent effect of C18:1AC on human vEHT and aEHT contractility (time course raw data).** Beating rate (left panel) and contractile force (right panel) of human **(a)** vEHT and **(b)** aEHT exposed to two different concentrations of C18:1AC for 3 days. Note time-dependent impairment of contractility at 25 µM of C18:1AC in both models. EHTs exposed to solvent only or to the short-chain AC served as controls. **(c)** Pre-treatment of aEHT with 1 µM of C18:1AC for 3 days as indicated before exposure to 25 µM did not protect from C18:1 induced force decline. Numbers given as total number of EHTs/number of batches. *p<0.05, **p<0.01, mean ± SEM.


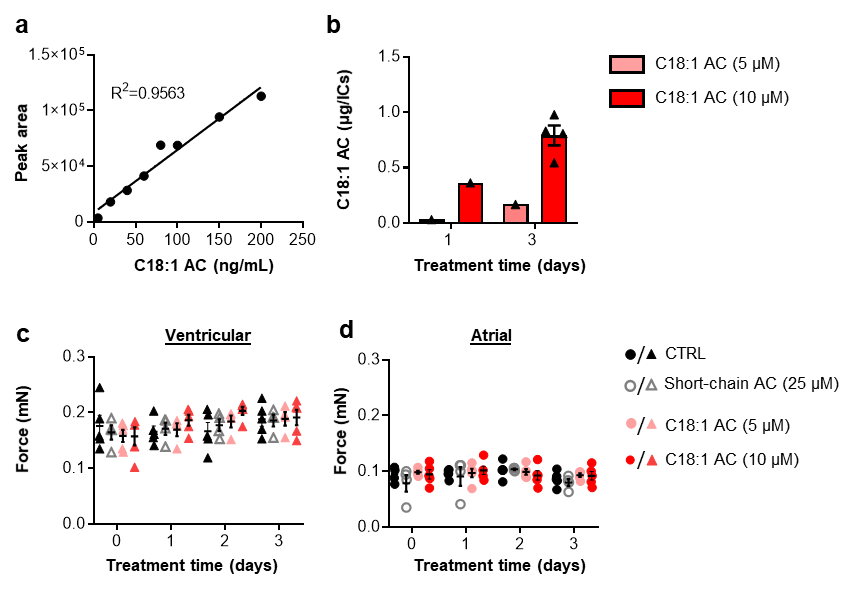


**Additional file 1: Fig. S12. Detection of C18:1AC in human EHT. (a)** Calibration curve for C18:1AC detection within a range of 5-500 ng/mL using HPLC-ESI-MS/MS. **(b)** Accumulation of C18:1AC in ventricular hiPSC-derived cardiomyocytes dissociated from one EHT each (per dissociated EHT: 1 million cells) over a period of 3 days (n=1-4). Force of human **(c)** vEHT and **(d)** aEHT in response to exposure to solvent only (vEHT n=5/1, aEHT n=5/1), 25 µM short-chain AC (vEHT n=4/1, aEHT n=4/1), 5 µM C18:1AC (vEHT n=4/1, aEHT n=5/1) and 10 µM C18:1AC (vEHT n=4/1, aEHT n=5/1) over 3 days.


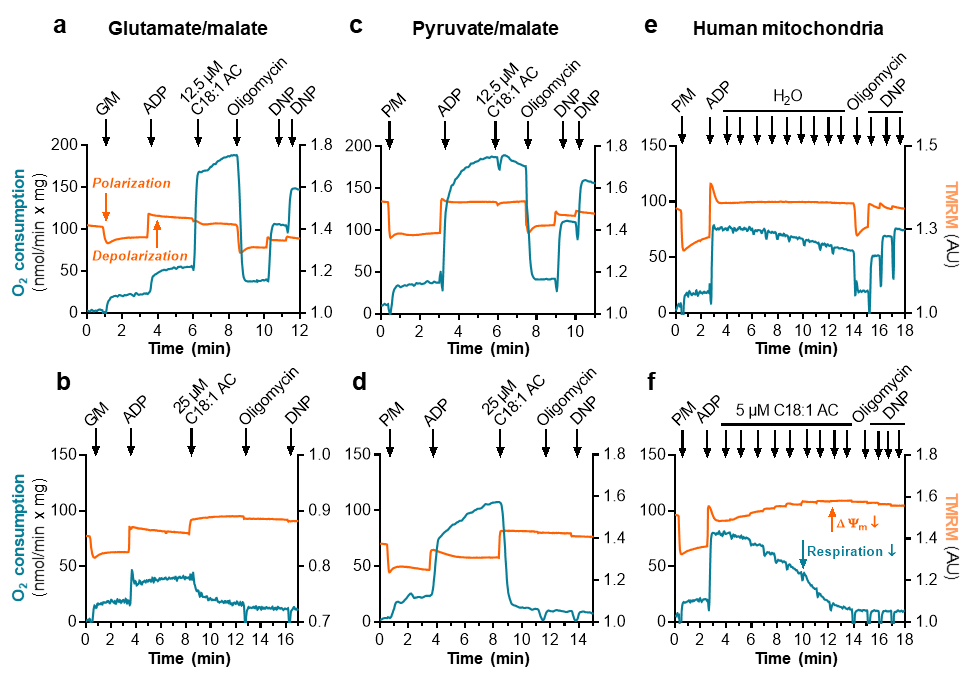


**Additional file 1: Fig. S13. Biphasic effect of C18:1AC on mitochondrial respiration.** Representative traces of mitochondrial respiration (blue) and membrane potential (TMRM fluorescence, orange) from murine cardiac mitochondria supplied with **(a-b)** glutamate/malate (G/M) or **(c-d)** pyruvate/malate (P/M) as substrates, exposed to 12.5 or 25 µM C18:1AC. Consecutive addition of **(e)** solvent control and **(f)** C18:1AC (5 µM each) during P/M respiration of human atrial mitochondria. AU=arbitrary units, ADP=adenosine diphosphate, DNP=dinitrophenol, TMRM=tetramethylrhodamine methyl ester.


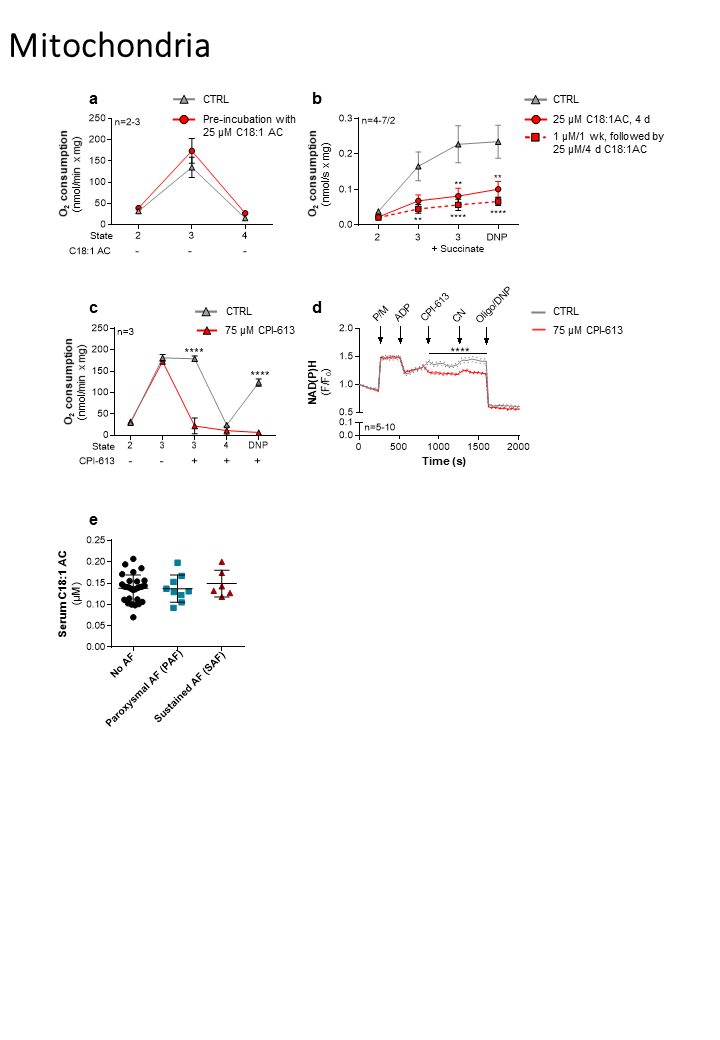


**Additional file 1: Fig. S14. Influence of C18:1AC on mitochondrial respiration**. **(a)** After 5 min of pre-incubation with C18:1AC (25 µM), mitochondria were resuspended in AC-free respiration buffer (n=2-3 mouse hearts, 400 µg of mitochondria per experiment). Mitochondria were supplied with pyruvate/malate (P/M) as substrate (state 2), and respiration was stimulated with saturating ADP (1 mM, state 3). Finally, oligomycin (1.2 µM) was added to block the F_1_-F_o_ ATP-synthase (state 4). Pre-incubation with AC did not affect respiration, excluding irreversible damage to mitochondrial membranes. **(b)** Mitochondrial respiration in intact aEHT (4-7 per group from 2 batches), which had been exposed to either solvent only (CTRL), to 25 µM C18:1AC for 4 days or to 25 µM C18:1AC for 4 days, after pre-treatment with 1 µM C18:1AC for 3 days. State 2: P/M/G as substrate. Succinate was added to probe additional respiratory chain complex II activity. Two-way ANOVA plus Bonferroni’s post-test for multiple comparisons, **p<0.01, ****p<0.0001, mean ± SEM. Effects of pyruvate dehydrogenase inhibition with CPI-613 (75 µM) on mitochondrial respiration **(c)** and NAD(P)H **(d)** of murine cardiac mitochondria supplied with pyruvate/malate (P/M) as substrate (state 2). CTRL indicated additional treatment with vehicle only. In **(d)**, cyanide (CN) was added to inhibit complex IV, thereby reducing NAD(P)H by preventing its oxidation by the electron transport chain; subsequently, oligomycin and DNP were added to induce complete oxidation of NAD(P)H. DNP=dinitrophenol. **(e)** Serum concentrations of patients from the Würzburg cohort whose mitochondria were used for mitochondrial respiration experiments in Figure 5. Mean ± SEM, one-way ANOVA plus Bonferroni’s post-test for multiple comparisons vs. No AF; no significant differences between groups.

**References**

1. Breckwoldt K, Letuffe-Breniere D, Mannhardt I, Schulze T, Ulmer B, Werner T, et al. Differentiation of cardiomyocytes and generation of human engineered heart tissue. Nat Protoc. 2017;12(6):1177-97.

2. Lemme M, Ulmer BM, Lemoine MD, Zech ATL, Flenner F, Ravens U, et al. Atrial-like Engineered Heart Tissue: An In Vitro Model of the Human Atrium. Stem Cell Reports. 2018;11(6):1378-90.

3. Hansen A, Eder A, Bonstrup M, Flato M, Mewe M, Schaaf S, et al. Development of a drug screening platform based on engineered heart tissue. Circ Res. 2010;107(1):35-44.

4. Hirt MN, Boeddinghaus J, Mitchell A, Schaaf S, Bornchen C, Muller C, et al. Functional improvement and maturation of rat and human engineered heart tissue by chronic electrical stimulation. J Mol Cell Cardiol. 2014;74:151-61.

5. Babakr AA, Fomison-Nurse IC, van Hout I, Aitken-Buck HM, Sugunesegran R, Davis PJ, et al. Acute interaction between human epicardial adipose tissue and human atrial myocardium induces arrhythmic susceptibility. Am J Physiol Endocrinol Metab. 2020;318(2):E164-E72.

6. Nickel AG, von Hardenberg A, Hohl M, Löffler JR, Kohlhaas M, Becker J, et al. Reversal of Mitochondrial Transhydrogenase Causes Oxidative Stress in Heart Failure. Cell Metab. 2015;22(3):472-84.

7. Nickel AG, Kohlhaas M, Bertero E, Wilhelm D, Wagner M, Sequeira V, et al. CaMKII does not control mitochondrial Ca(2+) uptake in cardiac myocytes. J Physiol. 2020;598(7):1361-76.

8. Kohlhaas M, Nickel AG, Bergem S, Casadei B, Laufs U, Maack C. Endogenous nitric oxide formation in cardiac myocytes does not control respiration during beta-adrenergic stimulation. J Physiol. 2017;595(12):3781-98.

9. Kirchhof P, Benussi S, Kotecha D, Ahlsson A, Atar D, Casadei B, et al. 2016 ESC Guidelines for the management of atrial fibrillation developed in collaboration with EACTS. Eur Heart J. 2016;37(38):2893-962.

10. Mela L, Seitz S. Isolation of mitochondria with emphasis on heart mitochondria from small amounts of tissue. Methods Enzymol. 1979;55:39-46.

11. Zeller T, Hughes M, Tuovinen T, Schillert A, Conrads-Frank A, Ruijter H, et al. BiomarCaRE: rationale and design of the European BiomarCaRE project including 300,000 participants from 13 European countries. Eur J Epidemiol. 2014;29(10):777-90.

12. Cavus E, Karakas M, Ojeda FM, Kontto J, Veronesi G, Ferrario MM, et al. Association of Circulating Metabolites With Risk of Coronary Heart Disease in a European Population: Results From the Biomarkers for Cardiovascular Risk Assessment in Europe (BiomarCaRE) Consortium. JAMA Cardiol. 2019;4(12):1270-9.

13. Kulathinal S, Karvanen J, Saarela O, Kuulasmaa K. Case-cohort design in practice - experiences from the MORGAM Project. Epidemiol Perspect Innov. 2007;4:15.

14. Gao X, Starmer J, Martin ER. A multiple testing correction method for genetic association studies using correlated single nucleotide polymorphisms. Genet Epidemiol. 2008;32(4):361-9.

15. R Core Team. R: A language and environment for statistical computing. R Foundation for Statistical Computing, Vienna, Austria. 2018.
